# Ultrahigh-throughput discovery of promiscuous enzymes by picodroplet functional metagenomics

**DOI:** 10.1038/ncomms10008

**Published:** 2015-12-07

**Authors:** Pierre-Yves Colin, Balint Kintses, Fabrice Gielen, Charlotte M. Miton, Gerhard Fischer, Mark F. Mohamed, Marko Hyvönen, Diego P. Morgavi, Dick B Janssen, Florian Hollfelder

**Affiliations:** 1 Department of Biochemistry, University of Cambridge, 80 Tennis Court Road, Cambridge CB2 1GA, UK; 2 INRA, UMR1213 Herbivores, F-63122 Saint-Genès-Champanelle, France; 3 Clermont Université, VetAgro Sup, UMR Herbivores, BP 10448, F-63000 Clermont-Ferrand, France; 4 Groningen Biomolecular Science and Biotechnology Institute, University of Groningen, Nijenborgh 4, 9747 AG, Groningen, The Netherlands

## Abstract

Unculturable bacterial communities provide a rich source of biocatalysts, but their experimental discovery by functional metagenomics is difficult, because the odds are stacked against the experimentor. Here we demonstrate functional screening of a million-membered metagenomic library in microfluidic picolitre droplet compartments. Using bait substrates, new hydrolases for sulfate monoesters and phosphotriesters were identified, mostly based on promiscuous activities presumed not to be under selection pressure. Spanning three protein superfamilies, these break new ground in sequence space: promiscuity now connects enzymes with only distantly related sequences. Most hits could not have been predicted by sequence analysis, because the desired activities have never been ascribed to similar sequences, showing how this approach complements bioinformatic harvesting of metagenomic sequencing data. Functional screening of a library of unprecedented size with excellent assay sensitivity has been instrumental in identifying rare genes constituting catalytically versatile hubs in sequence space as potential starting points for the acquisition of new functions.

## Abstract (web summary)

Environmental DNA from unculturable microorganisms contains genes with useful functions that remain difficult to identify and isolate. Here Colin, Kintses *et al.* demonstrate the screening of millions of samples in pL volumes to directly identify new enzymatic activities and complements sequence-based approaches.

## Graphical abstract

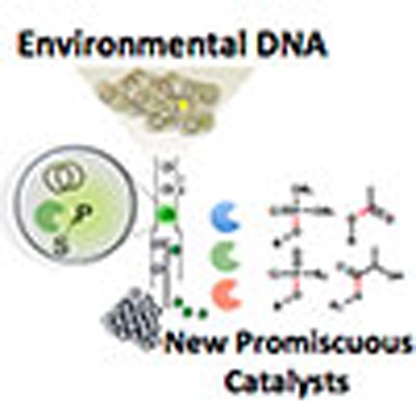

A much broader range of enzyme catalysts than currently known is needed to address a number of challenges—from the implementation of green, sustainable processes in white biotechnology^1^ to a fundamental understanding of the evolutionary origin and mechanistic basis of biocatalysis. Microbial ecosystems are viewed as enormous reservoirs of genetic diversity^2^, but 99% of environmental microorganisms are understood to be unculturable as yet^3^. Extensive sequencing efforts using DNA that was directly extracted from environmental samples (eDNA) have already given unprecedented insight into the make-up and genetic diversity of such ecosystems^4^. However, extrapolation from sequence to protein function is not trivial and often gives only a rough idea of functional assignments^5^. As a consequence, annotations frequently prove to be incorrect when tested experimentally^6^. Large-scale experimental characterization would be the preferred basis of functional annotation of proteins, but is currently time-consuming and expensive: which is why sequencing dominates metagenomic explorations. Furthermore, it is especially difficult to predict entirely new enzymes without precedent: genes without significant homology, encoding catalysts for unfamiliar reactions that have not yet been comprehensively assigned are likely to be overlooked (or remain unannotated). As a consequence, the percentage of genes with unknown functions in newly sequenced genomes has remained constant over the last decade (at ∼30–40%)^7^.

Functional annotation is further complicated by increasing evidence for catalytic promiscuity (that is, the ability of enzymes to process more than one type of substrate). Promiscuous side activities assist evolutionary adaptation by providing a head start activity after gene duplication, enabling a smoother route from one enzyme function to another by avoiding loss of function during their interconversion^8^,^9^. Enzyme promiscuity is even harder (and often impossible) to predict by sequence analysis than primary activities^10^, thus adding multiple dimensions to the challenge of experimental identification of function. As a consequence, the functional potential of this ‘underground network'^11^ remains largely unexplored. Functional characterizations of protein families suggest however that promiscuity is an intrinsic enzyme property^12^,^13^, a potential marker for evolutionary related proteins^14^ and a rich source of new functions^15^. Apart from chance observations no comprehensive system for detection of promiscuity exists. Discovery of promiscuous activities will be valuable: by analogy to natural neofunctionalization based on promiscuity, the identification of promiscuous activities similarly provides starting points for enzyme repurposing by directed evolution and can ultimately yield useful catalysts of practical utility.

Experimental screening of eDNA, where the corresponding proteins are heterologously expressed in a surrogate host, is a powerful method to functionally identify and annotate novel enzymes without relying on homology^16^. However, success depends on the efficiency of the heterologous expression^17^ and hit rates are typically extremely low (estimated at one hit per 10^4^–10^5^ variants, depending on the target activity)^18^. Efficient ultrahigh-throughput systems are therefore essential to cover enough eDNA sequence space to beat the odds^19^. Apart from costly robotic screening of individual metagenomic library members, requiring expensive liquid handling systems^20^ and labour-intensive procedures, no direct screening system for catalytic product formation (and able to screen large libraries to yield large numbers of hits) has been used to isolate novel catalysts in functional metagenomics. Apart from providing ultrahigh throughput, an experimental system for identifying promiscuous activities in metagenomes must be sensitive enough to pick up potentially weak side activities, as their rates are often several orders of magnitude slower than native activities^15^.

To address the challenge of performing sensitive functional screening with ultrahigh throughput, we use water-in-oil droplets, a biomimetic compartment acting as a genotype–phenotype linkage^21^,^22^, in analogy to cells. Monodisperse droplets are produced in microfluidic chips so that quantitative and sensitive measurements can be performed^23^ and ultrahigh-throughput sorting^24^ enables selection of droplets according to their fluorescence. Using this format, up to ∼10^8^ biochemical reactions can be performed per day, typically in pico- to femtolitre volumes, resulting in dramatically lower screening costs compared with robotic screening^25^. Microfluidic droplets have been successfully used for directed evolution^25^,^26^,^27^ strain selections^28^,^29^ or bioprospecting^30^. We now employ such droplets as vessels to miniaturize cell lysate assays^27^—the most frequently used screening formats in metagenomics^19^,^31^—to the single-cell level. In this experimental set-up, each library member is represented by a single cell, statistically compartmentalized into one droplet and assayed for catalytic activity after cell lysis. A microfluidic screening platform is used to recover and identify various hydrolases from metagenomic sources by screening for the hydrolysis of two substrates: sulfate monoesters, containing one of the most unreactive functional groups in biology^32^, and phosphate triesters. The latter are non-natural environmental pollutants historically used as pesticides^33^ that we now use to probe the catalytic potential of microbial communities for degradation of xenobiotics. The high sensitivity of our platform enabled us to isolate enzymes with strong and weak activities, including promiscuous side activities that have not been selected in the course of evolution and could not be predicted by sequence homology.

## Results

### Screening for new hydrolases in microfluidic droplets

**Figure 1:**
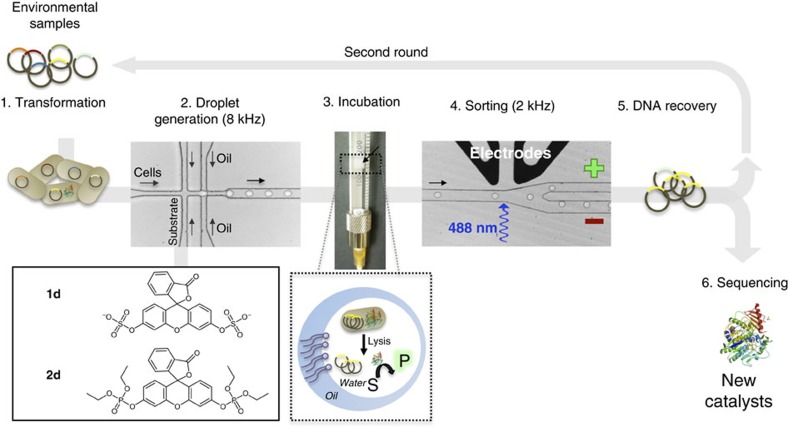
Functional metagenomic using microfluidic droplets. General procedure. (1) Environmental DNA (eDNA) was cloned into a high-copy plasmid and transformed into *E. coli.* (2) Single bacteria were encapsulated into water-in-oil droplets together with substrate (**1d** or **2d**) and lysis agents. (3) Emulsion droplets were incubated off-chip; after single cell lysis, cytoplasmically expressed protein catalysts were able to turn over substrate. The arrow designates the droplets (∼3 × 10^7^) at the interface between fluorous oil and mineral oil (on top). (4) Emulsion droplets were re-injected (Supplementary Movie 1) into a sorting chip and strongly fluorescent droplets (‘+' channel) were separated from those with fluorescence below the threshold (‘−' channel) by dielectrophoresis^24^ (Supplementary Movie 2). (5) Selected droplets were de-emulsified and high-copy plasmid DNA was recovered following by re-transformation into *E. coli*. For further enrichment, iterative selections could be performed. (6) Plasmids containing eDNA coding for active catalysts were sequenced. The microfluidic devices are shown in Supplementary Fig. 5.

**Figure 2:**
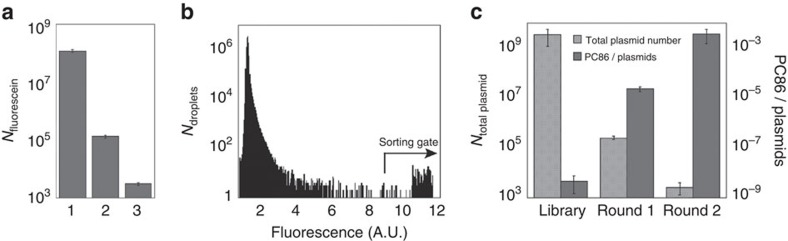
High sensitivity of the microfluidic platform allows selection and enrichment of active metagenomic variants. (**a**) Minimal number of fluorescein molecules detected. (1) In a 200 μl well of a microtiter plate (conventional technology), (2) in 20 pl droplets^27^ and (3) in 2 pl droplets (according to respective calibration curves; Supplementary Fig. 2). Errors bars are set to 10% of the calculated value, corresponding to an estimation of the uncertainty of measurement. (**b**) Fluorescence signal distribution of 10^7^ droplets containing metagenomic cell lysate after 2 days of incubation at 22 °C. The sorting gate was set up such that droplets with two- to fivefold increased fluorescence over the population average were selected (sorting gate). All histograms corresponding to the two screening campaigns are shown in Supplementary Fig. 7. (Note: as the photomultiplier tube saturated at around 10 AU, values shown here as ∼10 AU may be higher). (**c**) The selection stringency was tested by monitoring the enrichment of a PTE variant PC86 over multiple rounds of microfluidic sorting. Three samples—metagenomic library before selection; DNA recovered from round 1; DNA recovered from round 2—were analysed for (i) their total plasmid content and (ii) the number of plasmids encoding PC86 by quantitative PCR. The proportion of PC86 in each DNA sample (library before selection, round 1 and round 2) was calculated by dividing the number of plasmid PC86 by the total number of plasmids. (Detailed data are shown in Supplementary Fig. 10). Error bars, s.d. from triplicates. AU, arbitrary units.

**Figure 3:**
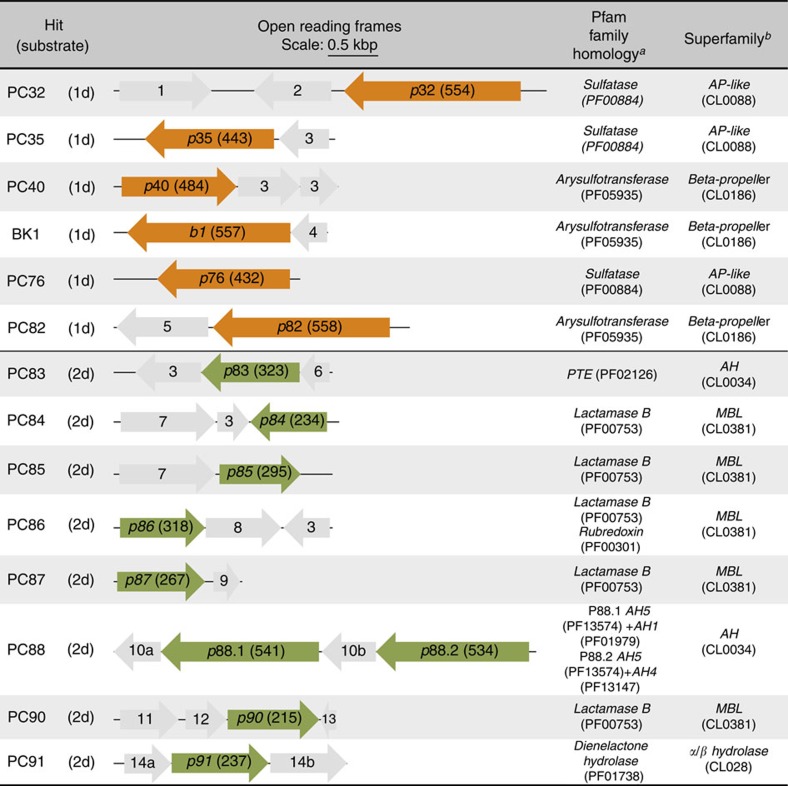
Unique hits isolated from metagenomic libraries. Open reading frames (ORFs) encoding hits are highlighted in orange or green (sulfate hydrolases/transferases or PTEs, respectively). Numbers between brackets indicate the number of amino acids in the protein sequence. Grey arrows represent other ORFs isolated together with the hit sequence. As it was less obvious which of the *p*88.1 or *p*88.2 gene was most likely to code for the active triesterase, both were recloned. Selections were carried out with either sulfate ester **1d** or phosphate triester **2d**. (a,b) Data extracted from the Pfam database^36^. MBL, metallo *β*-lactamase, also called metallo-hydrolase/oxidoreductase; AH, amidohydrolase. The other genes isolated (grey arrows) have their closest homologues in the NCBI non-redundant database predicted as: (1) transcription regulator; (2) formylglycine generating enzyme; (3) ABC transporter or membrane protein; (4) TonB-dependent receptor; (5) succinate-semialdehyde dehydrogenase; (6) carnitine dehydratase; (7) penicillin-binding protein; (8) radical SAM protein; (9) K^+^/H^+^ antiporter; (10a,b) cobalamin biosynthesis protein; (11) YKuD transpeptidase; (12) peptidase M15; (13) aminotransferase; (14a,b) permease (see Supplementary Note 1).

A metagenomic library of 1,250,000 variants pooled from a variety of sources (combining libraries derived from soil, degraded plant material and cow rumen samples; ‘SCV', Supplementary Table 1) was screened in droplets following the strategy illustrated in Fig. 1. Library members were expressed in *Escherichia coli* and single cells were compartmentalized (with a Poisson distribution that gives an average of ∼0.8 cells per droplet) into monodisperse 2 pl droplets (a volume found to allow suitably sensitive screening of metagenomic libraries—Fig. 2a and Supplementary Figs 1 and 2). Bacteria were co-compartmentalized with lysis agents and the respective fluorogenic substrates (sulfate monoester **1d** or phosphate triester **2d**; Fig. 1) in two different screening experiments. After a two-day off-chip incubation, the emulsions were re-injected into the sorting chip, where ∼20 million droplets (covering the library size more than 15 times and found to be sufficient to recover all hits in a model library; Supplementary Figs 3 and 4) were analysed in ∼2 h and the brightest 0.01% (sulfatases) or 0.01 and 0.001% (phosphotriesterases (PTEs), in two sorts performed in parallel and later combined) of the droplets selected (Fig. 2b). This relatively tolerant detection threshold was chosen to avoid loss of potential hits. The selected droplets were subsequently de-emulsified and plasmid DNA was recovered. The high-copy plasmid DNA allowed its direct re-transformation into *E. coli*^27^. The final number of transformants exceeded 10^4^ (one droplet gave on average approximately five transforming clones depending on the plasmid copy number found to vary as a function of the insert size; Supplementary Fig. 6). To enrich the hits further, a second microfluidic screening cycle (Fig. 1) was performed with a lower cell/droplet ratio (0.1 cells per droplet), reducing the number of droplets containing multiple cells. Out of ∼10 million screened, ∼500 droplets were collected as arylsulfatase hits and ∼300 as PTEs (corresponding to ∼0.007% or ∼0.003% of droplets for sulfatases and PTEs, respectively; Supplementary Figs 7 and 8). The resulting transformants were screened on Petri dishes or in 96-well plates for hydrolysis of sulfate monoester **1c** or phosphate triester **2d**, respectively. In 10% of the variants hydrolytic turnover was confirmed (Supplementary Fig. 9). Sequencing the plasmids of the confirmed positive hits revealed six and eight unique sequences for sulfatases and PTEs, respectively (Fig. 3). The low positive/negative ratio observed at the end of the screening campaign can be explained mainly by the deliberately tolerant sorting gate chosen to avoid loss of relevant clones at the cost of collecting false positives: droplets with a two- to fivefold higher fluorescence than background were selected (Fig. 2b and Supplementary Fig. 7).

### Assessing enrichment over microfluidic rounds

Despite the permissive sorting gate applied (Fig. 2b and Supplementary Fig. 7), we sought to quantify the enrichment obtained in our screening campaign. To this end, the abundance of one PTE hit in the library (PC86; Fig. 3) was analysed using quantitative PCR before and after the two rounds of sorting. Figure 2c shows that the plasmid content of this hit in the library increased >1,000-fold during the first round and >100-fold during the second round of sorting, corresponding to an overall enrichment of >10^5^ (from 10^−6^% to nearly 0.5%; Supplementary Fig. 10). Such enrichment capacity betters previously reported hit rates in functional metagenomic selections (estimated to be between 1/10,000 and 1/100,000)^18^, demonstrating the utility of the microdroplet format to recover extremely rare hits. An enrichment of more than 1,000-fold in one round of screening (despite a permissive sorting gate) is similar to or exceeds other studies using microfluidic droplets^24^,^27^,^34^. Sorting with greater stringency could be applied to achieve a similar enrichment in fewer rounds^35^. The objective of extending coverage to include hits even with weak activity, however, was made possible by a low detection threshold (<3,000 fluorescein molecules being detected; Fig. 2a and Supplementary Fig. 2) and exemplified the ability to detect low activities (Supplementary Fig. 11).

### Characterization of metagenomic hits

(a) *Sulfatase hits*. Our screening workflow yielded six unique variants (Fig. 3) that turned over sulfate ester **1d** (Fig. 1). Three hits possessed genes predicted to be part of the sulfatase family (see Fig. 3 for the assignments by Pfam, the protein families database^36^). One gene (*p*35) was recloned, the protein P35 was purified and shown to hydrolyse sulfate ester **1a** (Supplementary Fig. 12) with high efficiency (*k*_cat_*/K*_M_=1.7 × 10^5^ s^−1^ M^−1^; Supplementary Table 3) and high proficiency (*k*_cat_/*K*_M_/*k*_uncat_=1.5 × 10^14^ M^−1^; Supplementary Table 4), demonstrating the ability to recover an enzyme with high specific activities from our metagenomic libraries. Three hits contained genes that were assigned to the Pfam arylsulfotransferase family (*p40*, *b1* and *p82*). We experimentally verified this assignment after recloning and purification of the protein P40 (Fig. 3). P40 was shown to catalyse sulfate transfer from sulfate ester **1a** to phenolate following a ping-pong mechanism (Supplementary Fig. 13) and can therefore be considered as a new adenosine 3-phosphate-5-phosphosulfate-independent sulfotransferase^37^. Reactions in droplets were followed by the release of the fluorescent leaving group of **1d**. Transferases able to transfer sulfate groups to acceptors present in the cell lysate were therefore also selected, indicating that multiple types of related reactions can be recovered by the fluorogenic miniaturized cell lysate assay.

*(b) PTE hits.* Applying the screening workflow to PTEs (using triester **2d** as substrate) led to the isolation of eight unique and novel sequences, mostly originating from unknown microorganisms (Supplementary Table 2). Only one of these genes (*p83*) was predicted as a potential PTE by Pfam domain recognition (Fig. 3). To confirm PTE activity, we identified the genes most likely to encode triesterase enzymes and cloned them into an expression vector. Nine genes in total (*p*83, *p*84, *p*85, *p*86, *p*87, *p88.1*, *p88.2*, *p90* and *p91*; highlighted in green in Fig. 3) were recloned and their encoded proteins purified. All enzymes were active towards the fluorogenic triester **2d** (Fig. 1) used for screening, confirming the isolation of genuine PTEs (Table 1). When tested with the pesticides paraoxon **2a** or parathion **2b** (Supplementary Fig. 12), 5- to 100-fold slower rates than for the fluorogenic triester **2d** were observed (8 M^−1^ s^−1^ for P84 to 3 × 10^3^ M^−1^ s^−1^ for P83; Table 1). All hits degraded the fluorogenic screening substrate and the pesticide, suggesting that triester **2d** is a suitable bait for identifying paraoxon hydrolases. Despite rather low second-order rates (8–3 × 10^3^ M^−1^ s^−1^), most of these enzymes substantially accelerated hydrolysis of **2a** with rate enhancements up to 10^12^ (*k*_cat_*/K*_M_*/k*_w_), when compared with the spontaneous hydrolysis in water (Supplementary Table 4).

### Access to unexplored sequence space

**Figure 4:**
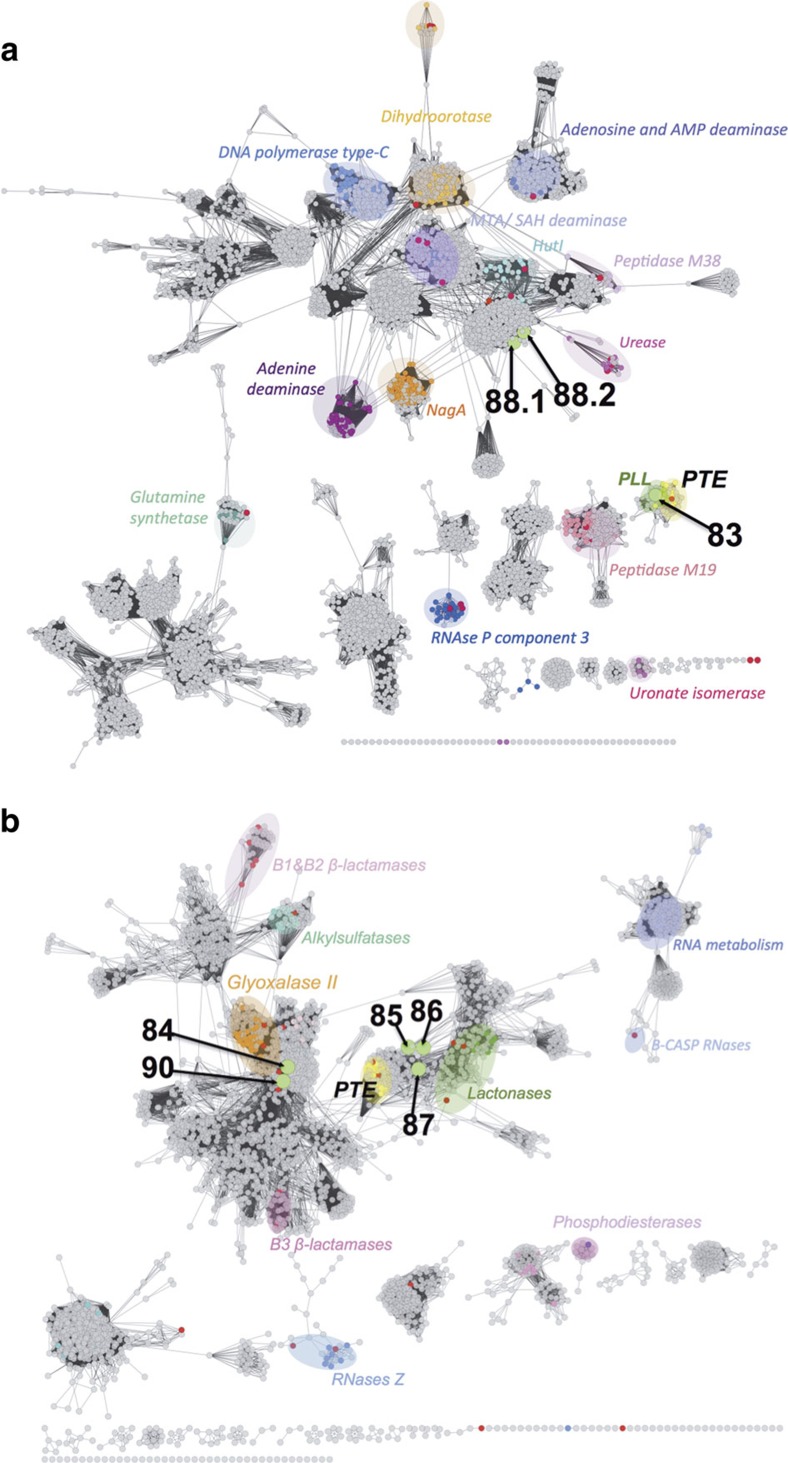
SSNs highlight the novelty of the triesterases hits. Hits are spread over (**a**) the AH clan (Pfam number: CL0034) and (**b**) the MBL superfamily (Pfam family PF00753). Bright green nodes represent the sequences of metagenomic hits identified in this work. Annotations retrieved from the Uniprot database were used to putatively characterize sequence clusters (represented by coloured nodes). To confirm these annotations, experimentally characterized proteins (red nodes) were added into each network. Previously described OP-degrading enzymes (PTE, phosphotriesterase) are highlighted in yellow; PLL (PTE-like lactonases) are reported in the AH superfamily. 5,042 and 2,984 sequences define the AH and the MBL networks, respectively. Edges lengths represent protein sequence similarity. Only edges corresponding to similarity scores below E-values of 1 × e^−9^ (AH) and 1 × e^−14^ (MBL) are considered; the worst edges displayed correspond to a median 26.5% (or 27.9%) identity over an alignment length of 210 (or 218) residues for the AH (and MBL) networks, respectively. See also the position of additional hits in the α/β superfamily (Supplementary Fig. 15).

Sequence-similarity networks^38^ (SSNs) (Fig. 4 and Supplementary Fig. 15) were constructed to map hit sequences: instead of being clustered together they were spread over three superfamilies (defined by the Pfam database^36^; Fig. 3) and covered a large sequence diversity. The only predicted triesterase (*p*83) located within the PTE /PTE-like lactonase cluster of the amidohydrolase superfamily (AH; Fig. 4a) and its higher activity towards lactone **8c** (Supplementary Fig. 12 and *k*_cat_*/K*_M_=5 × 10^4^ s^−1^M^−1^; Table 1) were verified experimentally, indicating that P83 is a new lactonase endowed with phosphotriesterase activity.

**Figure 5:**
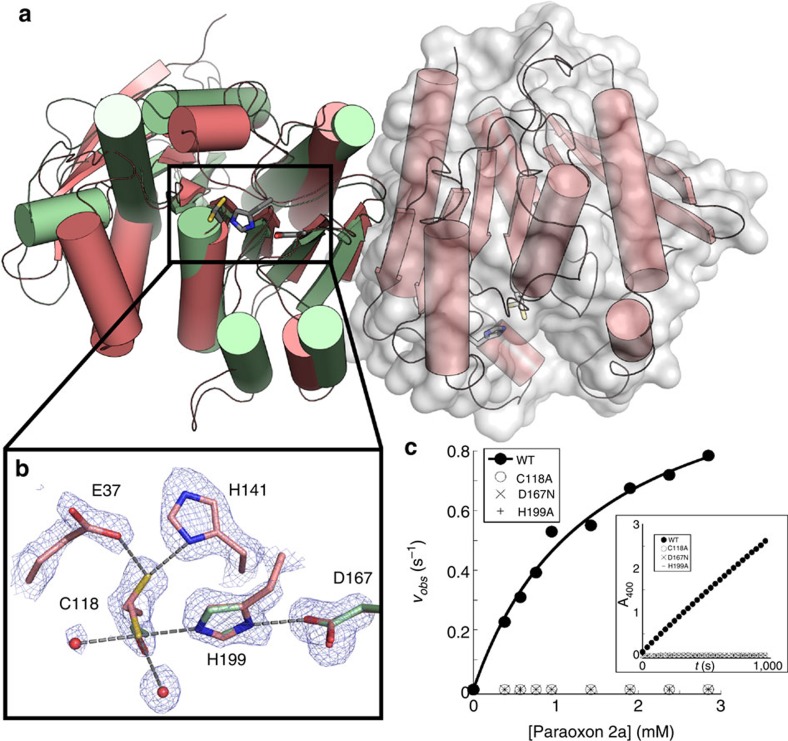
A triad is the catalytic feature of the α/β-hydrolase fold of the triesterase P91. (**a**) The P91 structure (red) is aligned with DLH (green), a well-characterized α/β hydrolase fold (PDB ID: 1ZIC^43^). (**b**) Active site superposition with DLH reveals the catalytic triad of P91 consisting of C118, D167 and H199. E37 and H141 stabilize an alternative cysteine conformation. (**c**) Site-directed knockdown mutagenesis of the residues in the triad compromises P91's PTE activity (*v*_*obs*_=*v*/[E_0_]) (shown by Michaelis–Menten plot and time-course measurements using ∼1 mM of substrate (framed)). Conditions: activity towards paraoxon **2a** measured at 25 °C (100 mM Tris-HCl, 150 mM NaCl pH 8.0).

The remaining hits defied prediction and were scattered over three superfamilies. (i) Two were also AH superfamily members (P88.1 and P88.2) but were located in a completely different region of the SSN (Fig. 4a) and shared very little sequence identity (<15%) with other PTEs from the same superfamily (Supplementary Fig. 16). No other native function was predicted, making them completely unassigned. (ii) All hits assigned to the metallo-β-lactamase (MBL) superfamily (P84, P85, P86, P87 and P90) have close homologues in the NCBI non-redundant database predicted as putative β-lactamases. However, none of the five hits was able to degrade the chromogenic β-lactam **10** (Supplementary Fig. 12), a frequently used probe of β-lactamase activity^14^,^39^. In the SSNs (Fig. 4b), the genes *p*85, *p*86 and *p*87 located in proximity to sequences coding for PTEs and lactonases. However, no lactonase activity was detected in any of these hits (with lactones **8a**, **8b**, **8c** and **8d**_**1–2**_; Supplementary Fig. 12), confirming that sequence similarity is not a good predictor for activity. P84 and P90 are positioned away from other known triesterases and seem more related to glyoxalase II (Fig. 4b), a family of thioesterases from the glyoxalase system that catalyses the hydrolysis of *S*-lactoylglutathione into glutathione and lactate^40^. Experimental verification (Table 1) of their ability to hydrolyse *S*-lactoylglutathione **7** (Supplementary Fig. 12) suggested that these enzymes were a subgroup of the glyoxalase II family. (iii) One hit, P91, is a member of the α/β-hydrolase (α/β) superfamily related to dienelactone hydrolases (DLHs; Fig. 3). Members of this superfamily possess a catalytic triad^41^, whereas most bacterial PTEs described to date use catalytic divalent ions to activate a water nucleophile^42^. The X-ray structure of the protein was determined at a resolution of 1.7 Å (Supplementary Fig. 18) and structural alignment with a previously characterized DLH^43^ (also identified in the SSNs, shown in Supplementary Fig. 15) confirmed an α/β hydrolase fold (Fig. 5a), and highlighted a homologous catalytic triad in the active site of P91 (C118, D167 and H199; Fig. 5b). Site-directed knockdown mutagenesis of C118, D167 and H199 led to enzyme inactivation, consistent with catalysis by the cysteine-based catalytic triad in P91 (Fig. 5c), which was expected to provide nucleophilic and charge relay catalysis. We observed electron density consistent with two orientations of C118. Figure 5b highlights potential ‘protecting' residues (E37 and H141) that may play a role in stabilizing an inactive conformation of the nucleophile cysteine, as previously invoked^44^. The lack of electron density in the vicinity of the active site supported the absence of a metal-binding site, underlining a role for the triad instead of a metal cofactor in catalysis. Functional tests further ruled out P91 as a metallo-enzyme: treatment of P91 with chelating agents did not affect its activity towards triester **2a**, whereas a reduction (or complete abolition) of activity was observed in all other PTE hits tested (Supplementary Fig. 17).

Acetylcholine esterases are the biological targets of organophosphate (OP) pesticides, acting as suicide substrates leading to acetylcholine esterase inactivation and belong to the α/β superfamily (see SSNs of the superfamily in Supplementary Fig. 15). It has been reported that α/β hydrolases have evolved in insects (for example, *Lucilla cuprina*) to slowly degrade OPs^45^, conferring insecticide resistance in the absence of a metal cofactor. However, P91 is the first metal-free bacterial (or archaeal) triesterase to be described, unrelated to insect carboxylesterases (Supplementary Fig. 15). Most of these carboxylesterases have evolved ageing-resistant variants^46^ and are characterized by low turnover rates combined with tight binding^45^. In contrast, P91 is a weak paraoxon binder (*K*_M_ ∼mM) but with relatively high turnover rates (∼1 s^−1^), suggesting that P91 does not suffer enzyme ageing, allowing up to four orders of magnitude higher *k*_cat_ (ref. 45).

### Selection of catalysts via a promiscuous function

**Figure 6:**
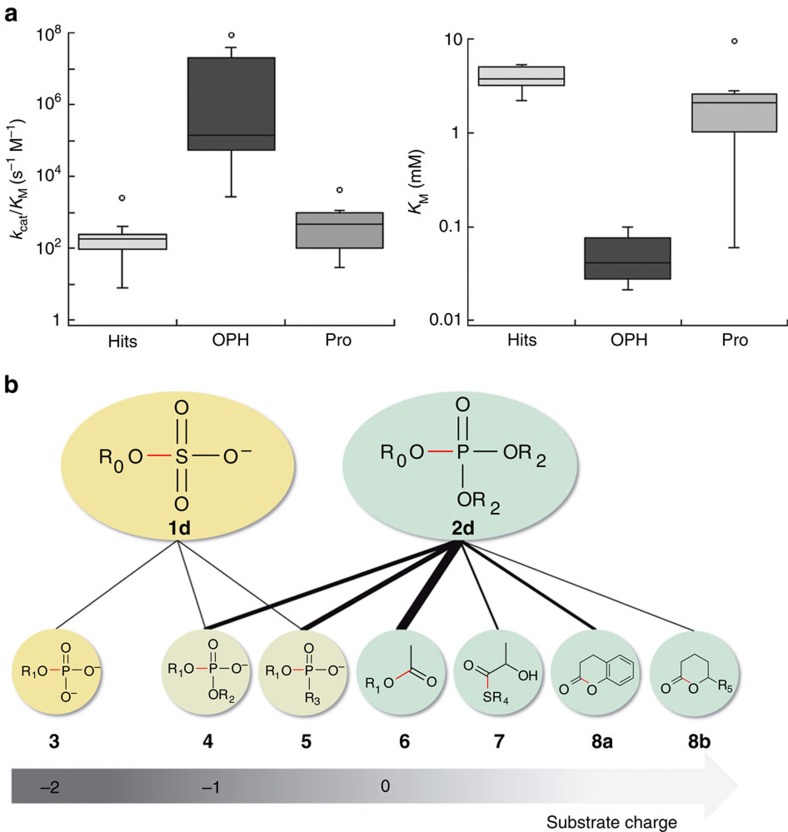
Promiscuity is a general feature of the selected hits. (**a**) Comparison of the data (catalytic efficiencies *k*_cat_/*K*_M_ (left) and *K*_M_ values (right)) for our hits with known enzymes catalysing phosphotriester hydrolysis suggests that our screening has identified promiscuous activities. ‘OPH' summarizes PTE recovered from bacteria isolated from a pesticide-polluted environment and therefore assigned as enzymes that evolved specifically for triester hydrolysis. ‘Pro' designates enzymes for which PTE activity was shown to be a promiscuous side activity. The rates reported here are towards paraoxon **2a** (for metagenomic hits) or—in the case of known PTEs—towards paraoxon **2a** or methylparathion **2b**. Full names (Supplementary Table 6) and reported catalytic efficiencies (Supplementary Table 7) of known PTEs are listed. (**b**) The catalytic promiscuity among the triesterase hits reflects bait substrate charge attributes. Substrates **1d** and **2d** are the bait substrates and substrates **3**–**8c** were used for catalytic profiling. The likely bonds cleaved are represented in red. Black lines indicate that the hits share these two activities. Their width represents how often this type of enzyme promiscuity was observed. The *x* axis groups substrates by their charges and suggests that the bait activities select hits with consistent promiscuous activity patterns in which substrate charge is a key determinant. *R*_0_=fluorescein; *R*_1_=4-nitrophenyl; *R*_2_=ethyl; *R*_3_=methyl; *R*_4_=glutathione (which has two additional negative charges remote from the reaction centre) *R*_5_=C_6_H_13_. See Supplementary Fig. 12 for structures of all substrates.

The comparison of the Michaelis–Menten parameters (*k*_cat_*/K*_M_ and *K*_M_) of our triesterase hits with those of (i) OP hydrolases isolated from pesticide-contaminated soils and (ii) enzymes with known promiscuous triesterase activity (Fig. 6a and Supplementary Table 5) suggests that paraoxon **2a** (or parathion **2b** with lower or similar catalytic rates measured; Supplementary Table 3) and, by extension, the synthetic triester **2d** are not the physiological substrates of our hits. This conclusion was also supported by the experimental verification of higher activities in P83, P84, P90 and P91 towards lactone **8c**, thioester **7** and ester **6**, respectively (Table 1 and Supplementary Table 3).

This observation provides further evidence for the widespread existence of latent enzymatic activities, enabling biocatalysts to promote ‘unnatural reactions' besides their native function. Most PTEs known to date were isolated from OP-degrading bacteria, providing a selective advantage in highly OP contaminated soils^47^. As OP-based pesticides were only introduced into the environment in the 1940s (ref. 42), these enzymes are examples of rapid evolutionary adaptation. Our screens suggest the availability of additional points for ‘head starts' in adaptive evolution, spread over three superfamilies. Previously, only ∼20 experimentally characterized PTEs were reported in the literature (Supplementary Table 6). We now have increased this number by a third and doubled the number of enzymes with promiscuous triesterase activity.

Many PTEs seem to have evolved from lactonases and retain some degree of lactonase activity^14^,^48^,^49^. However, most of our hits were not active (Table 1) towards the natural lactones tested (**8b**, **8c** and **8d**_**1–2**_), suggesting that they have different evolutionary ancestors than other known PTEs (also supported by their position in the SSNs in Fig. 4 and Supplementary Fig. 15) and implying that diverse native activities can accommodate promiscuous triesterase function. As the hits locate mostly in sparsely annotated regions in their respective SSNs (Fig. 4) and because of the relatively short insert sizes, only limited additional information is provided by the genome context (Fig. 3 and Supplementary Note 1), the native activities and physiological roles of the hits remain unknown.

The majority of the triesterase hits constitute first examples of enzymes endowed with triesterase activity within their respective superfamily subgroup underlining that a different region of sequence space is accessible by droplet screening compared with metagenome mining by bioinformatics. Accessing such new isolated but functionally characterized points in sequence space makes them hubs for exploration of close homologous sequences that may share similar activities^50^.

### Novel entry points to promiscuous networks

The two bait reactions (that is, phosphotriester hydrolysis and arylsulfate hydrolysis) unearthed six different activities in total: sulfate monoesterase, phosphate monoesterase, phosphate diesterase, PTE, phosphonate monoesterase and (thio)-esterase activities (Fig. 6b). Promiscuity emerged as a feature of all hits, with more than two chemically distinct substrates turned over besides the mostly unknown native ones, confirming the insight that promiscuity is ubiquitous^15^ and worthwhile harvesting. Many reactions were common among the hits despite their extreme sequence divergence (Supplementary Fig. 16) and their different catalytic machineries (Fig. 5b and Supplementary Fig. 17). A comparison of the observed promiscuity suggests trends that reflect the respective bait: sulfatases favour charged substrates, triesterases neutral ones. Phosphonate hydrolase and phosphodiesterase activities are common to the two groups (Fig. 6b), but triesterases support these activities only at a low level (Table 1). Testing a range of bait substrates in future experiments may lead to empirical cross-reactivity maps (similar to Fig. 6b) that describe active site recognition features (that is, geometric or electrostatic complementarity, or the availability of reactive active site groups, for example, nucleophiles).

## Discussion

One of the main limitations in functional metagenomics is the difficulty of discovering rare genes. Indeed, much work in this field has focused on widely occuring enzymes such as lipases, esterases and carbohydrate-converting enzymes^18^. To identify potentially less widespread activities, effective screening technologies are key^31^ to covering larger metagenomic sequence space^21^ and beating the odds. In this work, we establish droplet screening as a general method to find new, rare enzymes (6 and 8 hits out of 1,250,000 clones for sulfatase and PTE, respectively) in metagenomes using hydrolytic turnover of two different baits as initial target activities. Previous attempts at harvesting environmental samples by droplet screening have not successfully identified DNA sequences that correspond to the selected phenotype^30^. By contrast, this present work has yielded functional information on new metagenomic sequences from droplet experiments, for the first time. More generally, no metagenomic campaign had so far identified triesterases or sulfatases, possibly because they are much less abundant than the above-mentioned enzymes (and certainly with fewer known examples in the literature). Our screening capacity of >5 × 10^6^ assays per hour enables ∼10^8^ assays per day so that libraries of >10^6^ variants are easily accessible at low cost^25^ without relying on survival-based assays^51^ or substrate-induced regulators (for example, in SIGEX)^52^. The high sensitivity of the miniaturized cell lysate assay allowed the isolation of enzymes with catalytic efficiencies (*k*_cat_*/K*_M_) ranging from 54 M^−1^ s^−1^ (P84) to 9 × 10^5^ M^−1^ s^−1^ (P83; Table 1), providing evidence that this microfluidic platform is able to identify slow and fast catalysts, with a wide dynamic range extending over four orders of magnitude. Our protocol is straightforward as the readout is directly reporting on enzymatic activity, so that promiscuous activities are accessible. The versatility of microfluidic workflows^53^ makes our platform adaptable for different challenges: to further increase our assay sensitivity (or diminish incubation time) bacterial growth in droplets could be implemented after cell encapsulation, by implementation of an additional microfluidic injection (or droplet fusion) step to supply lysis agents and substrates. Furthermore, the microfluidic platform is readily applicable to other enzymatic activities, as long as a fluorogenic assay can be implemented.

Most (if not all) triesterase hits identified in this work must be presumed not to have evolved in response to an environmental OP triester challenge. Promiscuous activities can be recruited during evolution to give rise to new protein functions^8^,^9^,^15^. Our hits may therefore constitute alternative starting points for adaptive evolution in OP-contaminated environments giving rise to different OP-degrading enzymes than currently known. The systematic detection of such catalytic starting points can form the basis of evolutionary models that account for the adaptive potential of a microbial community to degrade exogenous compounds, such as pesticides and antibiotics. Likewise, directed evolution in the laboratory^10^ can use hits with low promiscuous activities as starting points that can be enhanced to generate new efficient catalysts.

In contrast to methods for active site mapping that rely on single turnover suicide inhibition^54^, the droplet format enables detection of multiple turnovers and indeed all catalysts selected show >10^3^ turnovers per enzyme molecule. As demonstrated here, weak or promiscuous activities are directly accessible by droplet screening. This is a powerful method to identify new enzymes for further improvement by protein engineering^55^ and also new entry points into networks of promiscuous activities^56^, for which there is currently no systematic prediction tool in bioinformatics. The choice of the bait substrate alone determines which catalytic processes are identified. The ultrahigh throughput and the high sensitivity of our method make microfluidic droplets a powerful format to explore this ‘promiscuome' (i.e. the collection of promiscuous activities from complex meta-proteomes), where other indirect methods fail. Additional optical detection modes and systematic exploration of libraries with assays for new substrates will be useful to exhaustively harvest many different classes of enzymes. Sensitive, fast and affordable microfluidic droplet screening is now validated as an alternative technology for enzyme discovery that can yield information not accessible by other approaches.

## Methods

### Metagenomic material

Environmental libraries from various soils^57^ and vanilla pods were constructed^58^ starting with shearing eDNA using a nebulizer and blunting it using Klenow fragment. Next, the resulting 3–5 kbp fragments were cloned into the EcoRV restriction site in the high-copy plasmid pZero-2 (Invitrogen). DNA from cow rumen was partially digested with the restriction enzyme MluCI. DNA fragments with sizes around 3 kbp were isolated by gel electrophoresis and cloned into the EcoRI site of the same high-copy plasmid pZero-2. The resulting ten libraries (Supplementary Table 1) were pooled together, constituting a library of about 1,250,000 variants, called the ‘SCV library'.

### Chip design and microfluidic device fabrication

The designs for the poly(dimethyl)siloxane (PDMS) chip devices were prepared using Autocad CAD software and designs are shown in Supplementary Fig. 4. The corresponding CAD files can be downloaded from http://openwetware.org/wiki/DropBase (a database of microfluidic device designs). The devices were fabricated with standard soft lithographic procedures^59^. The photoresist material SU-8 2015 was used to obtain a 15 μm channel height. PDMS monomer and curing agent were mixed at a ratio 10:1 and then poured onto the lithographic plate before degassing. After PDMS solidification (65 °C, 4 h), PDMS was activated by exposure to an oxygen plasma and devices were sealed onto a microscope glass slide (or cover slip (thickness: 0.13 mm) for the sorting chip). Hydrophobic modification of the channels surface was achieved by injecting a solution of 1% (v/v) trichloro(1H,1H,2H,2H-perfluorooctyl)silane (Sigma) in HFE-7500 oil into the channels. Electrodes for the sorting devices were prepared using low melting point indium composite solder (51 In/32.5 Bi/16.5 Sn, Indium Corporation).

### Preparation of bacterial suspensions for droplet encapsulation

Transformation of ∼10 ng of the metagenomic library SCV into *E. coli* (E. cloni 10G Elite, Lucigen) yielded ∼10^8^ variants on agar plate (containing 40 μg ml^−1^ kanamycin) covering the library size ∼100 times. Bacteria were grown overnight at 37 °C, then incubated at 22 °C for 2 days. Colonies were subsequently scraped from the agar plates, washed, filtered using a 5-μm filter (Sartorius) and resuspended in MOPS (100 mM, pH 8.0) containing NaCl (115 mM), kanamycin (40 μg ml^−1^), complete EDTA-free protease inhibitor (one tablet per 50 ml; Roche) and Percoll (25% v/v; Sigma). The cell density was adjusted by dilution to obtain the required cell/droplet ratio after compartmentalization. Assuming a Poisson distribution for bacterial encapsulation^60^, a cell density OD_600 nm_ ∼1 should result in ∼35% of droplets with single cells and ∼20% of droplets with higher occupancy (for φ 15 μm droplets).

### Generation of water-in-oil picolitre droplets

Water-in-oil droplets (volume ∼2 pl, φ: 15 μm) were generated using a flow-focusing device (Fig. 1) (dimensions width × height of 16 × 15 μm) bearing three inlets. Two inlets carry aqueous solutions prepared in MOPS buffer (100 mM, pH 8.0), NaCl (115 mM), kanamycin (40 μg ml^−1^), EDTA-free protease inhibitor (one tablet per 50 ml; Roche). The streams from these inlets supplied (i) a cell suspension (OD_600 nm_ depending on cell occupancy (i.e. number of cell per droplets) required) in Percoll (25%, v/v, Sigma) and (ii) a mixture of the cell lysis reagents BugBuster (20% v/v, Novagen) and lysozyme (30 kU ml^−1^; Novagen), as well as the respective substrate (10 μM of sulfate monoester **1d** or phosphate triester **2d**). From the third inlet, fluorinated oil HFE-7500 (3 M) containing EA surfactant (1%, w/w, RainDance Technologies) was injected. Aqueous solutions and the oil phase were injected using plastic syringes (BD; 1 or 3 ml) at a rate of 50 and 500 μl h^−1^, respectively, with PHD 2000 Harvard Apparatus pumps.

### Droplet storage and incubation

Droplets were stored in a glass syringe (500 μl, SGE) in HFE-7500 (3 M) with EA surfactant (1%, w/w), covered with mineral oil (Sigma). The fluorous phase containing droplets is not miscible with mineral oil and droplets remain at the interface between HFE-7500 and mineral oil. Droplets were incubated from 1 to up to 3 days in the syringe at room temperature (∼22 °C).

### Droplet sorting and electronics

After mounting the incubation syringe vertically on a syringe pump (PHD 2000 Harvard Apparatus), water-in-oil droplets were re-injected into the sorting device at a rate of 30 μl h^−1^. The distance between droplets was increased to facilitate the sorting of single droplets by electric pulses. To this end, additional fluorous oil (HFE-7500 (3 M) with EA surfactant (0.5%, w/w) was injected at 300 μl h^−1^ in the sorting chip. This set-up with a parallel channel design (with a width ratio of 1.3 between waste and positive channels, Supplementary Fig. 4) resulted in a droplet sorting rate of 2–2.5 kHz, without the need for pressure equilibration between the two outlets as previously required^27^. A 488-nm laser was focused 180 μm upstream of the sorting junction through a × 40 microscope objective (UPlanFLN, Olympus) for fluorophore excitation and the emitted fluorescent light was collected and amplified using photomultiplier tubes (H8249, Hamamatsu Photonics). The amplified fluorescence signal was processed by a data acquisition card operating at 38 kHz (National Instruments, USB-6009) that was linked to a peak detection algorithm, which recorded fluorescence distributions (LabView 8.2, National Instrument). Hardware triggering was implemented via a voltage comparator (LM339N, Texas Instruments), which compared the voltage readout by the photomultiplier tube with a user-defined arbitrary voltage generated via the acquisition card and doubled using an operational amplifier (LM358N, STMicroelectronics), to generate voltages between 0 and 10 V. A pull-up resistor (1 kΩ) was used to force the logical high state of the comparator to 5 V and send the trigger signal to the pulse generator. Whenever the fluorescence peak reached a user-defined voltage threshold (typically corresponding to two- to fivefold increase over the average fluorescence of droplets not containing active enzymes), a pulse generator triggered a single square pulse of 500 μs length and an amplitude of 0.6–0.8 V_p_. This pulse was amplified 1,000-fold by a high voltage amplifier (610E, Trek) and applied to the electrodes on the sorting device. With the current electronics implementation, 500 μs was found to be the minimal pulse length able to trigger the fast camera used as a control, to monitor the success of sorting. The electric pulse applied between the two electrodes dielectrophoretically attracted the highly fluorescent droplet towards the narrower channel (Fig. 1 and Supplementary Fig. 5). The sorting events were recorded with a fast camera (Phantom V7.2) that was triggered by the voltage comparator, to allow analysis of whether the desired droplets with increased fluorescence were selected. Optical inspection of the movies thus recorded monitored that only single droplets were selected for each pulse.

Detection of useful hits in this format was only possible after improvements to the sensitivity of the previously described miniaturized cell lysate assay^27^. This was achieved by adapting our microfluidic platform to generate and sort 2 pl droplet compartments (φ 15 μm). To test whether smaller droplets led to higher sensitivity, we measured the accumulation of fluorescein over time in two droplet populations with different volumes (2 and 8 pl) containing single bacteria transformed with a metagenomic variant active towards sulfate monoester **1d** (Fig. 1). Fluorescence signal change (average fluorescence of droplets containing bacteria divided by average fluorescence of empty droplets) increased approximately twice as fast in 2 pl droplets than in 8 pl droplets (Supplementary Fig. 1), confirming that sensitivity was improved by scaling down the droplet size.

### DNA recovery

Plasmid DNA from fluorescent droplets was recovered by de-emulsification using 1H,1H,2H,2H-perfluorooctanol^27^. The use of a high copy number plasmid (pZero-2; >1,000 copies per cell) in an *E. coli* endonuclease knockout strain (E. cloni 10G, Lucigen, bearing the mutation *endA1*) allowed plasmid DNA recovery from droplets even after 2 days of incubation, without additional PCR amplification.

### Quantification of enrichment of hits by quantitative PCR

To quantify the total number of plasmids from the library, a set of primers annealing to the vector was used:

5′-TTTCTGCGGACTGGCTTT-3′ (qPZeroFwd)

5′-ACAGGATTAGCAGAGCGAGG-3′ (qPZeroRev).

To quantify plasmids containing PC86 (as a representative metagenomic hit), primers annealing to the inserted PC86 sequence were designed:

5′-ATACCGACGAAGCCCTGT-3′ (qPC86Fwd)

5′-TCGGCAGGGTCATACACATA-3′ (qPC86Rev).

All primers were supplied by Invitrogen Life Technologies. Quantitative real-time PCR experiments (see also Supplementary Fig. 10) were performed in triplicate using Sensimix SYBR green (Bioline) in the Rotor-Gene 6000 (Corbett Life Sciences). The PCR conditions were as follows: initial DNA denaturation at 95 °C for 10 min, 40 cycles (95 °C for 10 s; 52 °C for 15 s; 72 °C for 20 s) and a temperature gradient enabling determination of the DNA melting temperature (between 72 °C and 95 °C). Reference curves using both sets of primers were obtained with correlation coefficients *R*^2^>0.99.

### Metagenomic screening on plates

Metagenomic libraries (ENR-S, ENR-G, ENR-M and ENR-L; Supplementary Table 1) were transformed into electrocompetent *E. coli* (E. cloni 10G Elite, Lucigen) and ∼100,000 clones were plated on 10 different φ 14 cm Petri dishes with Luria Bertani (LB) Agar (1.5%) containing 40 μg ml^−1^ kanamycin. After overnight growth at 37 °C, plates were incubated at 22 °C for 24 h. Colonies were then transferred onto nitrocellulose membranes (Pall Corporation) and lysed by three cycles of 10 min incubation at −20 °C and 37 °C before being overlaid with 100 mM Tris-HCl pH 8.0, 0.5% agarose (w/v) containing 135 μM of sulfate monoester **1c** (Supplementary Fig. 12). Colonies that turned blue after ∼15 h of incubation at 25 °C were isolated and their plasmid DNA extracted using a miniprep kit (Zymo Research) and sequenced.

### Fidelity of droplet sorting

We probed whether our miniaturized cell lysate screen in droplets was able to recover hits from metagenomic libraries. Thus, a subset of the library (∼100,000 clones) (Supplementary Table 1) was screened for sulfate hydrolases in two experiments: (i) in microfluidic droplets using sulfate monoester **1d** (>10^7^ droplets in 100 μl, protocol shown in Fig. 1) and (ii) in a classic colony screening procedure^61^ (400,000 colonies using 40 Petri dishes) using sulfate monoester **1c** as a substrate. When the two screens were compared, all hits found on plates were recovered within 0.5 h by droplet screening (Supplementary Figs 3 and 4), except one that was later isolated when the ENR-G library was screened alone. The ability of our system to detect and sort hits was further addressed by analysing the relationship between hit rate and sample size. When covering the library size with a variety of oversampling ratios (by screening a number of droplets corresponding to 2 × , 10 × , 20 × and 25 × the library size) we observed an increasing number of recovered model hits (Supplementary Fig. 4), suggesting that the increase in screening capacity indeed leads to more hits (assuming a library contains them). We found that a >10-fold oversampling of the library is sufficient to recover every hit confidently, thereby achieving perfect coverage (Supplementary Fig. 4).

### Protein production and purification

Open reading frames coding for sulfatase P35 and the sulfotransferase P40 were recloned into a modified expression vector pRSFDuet (Novagen) using the restriction sites NdeI–XhoI (P35) and NcoI–XhoI (P40). Recombinant plasmids pRSFDuetP35 and pRSFDuetP40 were transformed into *E. coli* BL21(DE3). For protein expression transformants were grown in 750 ml LB broth (containing 40 μg ml^−1^ kanamycin) at 37 °C until an OD_600 nm_∼0.5 was reached. At this stage, expression was induced with isopropyl-β-D-thiogalactoside (1 mM) for 15 h at 25 °C. Cells were harvested by centrifugation, resuspended in LB medium (30 ml) and lysed by sonication. Cell lysate was obtained by centrifugation (30,000*g*, 1 h, 4 °C) and diluted in a 1:1 ratio with Tris-HCl (50 mM, pH 8.0). All subsequent steps were carried out in this buffer, unless stated otherwise. The desired enzyme was purified from diluted cell lysates using a sequence of three columns as follows: (i) anion exchange (Q-sepharose Fast Flow; GE Healthcare) using NaCl gradients 0-400 mM (P35) or 0-1 M (P40), (ii) affinity chromatography (P-sepharose Fast Flow; GE Healthcare) using (NH_4_)_2_SO_4_ gradients 1-0 M (P35) or 500-0 M (P40), and (iii) gel filtration (Superdex 200) running in a Tris-HCl buffer (20 mM, pH 8.0). Chromatographic steps of the purification were carried out in a AKTA FPLC apparatus (GE Healthcare).

Open reading frames coding for PTEs were recloned into the pASKIBA5plus plasmid (Iba Life) using the BsaI restriction site (P83, P84, P85, P87, P881, P90 and P91) or EcoRI–NcoI restriction sites (P86 and P882). Recombinant plasmids were transformed by electroporation into *E. coli* BL21(DE3). Cells were grown at 37 °C in LB containing 100 μg ml^−1^ ampicillin (750 ml) until OD_600 nm_∼0.5. The expression of the amino-terminal Strep-tagged proteins was induced with anhydrotetracyclin (200 μg l^−1^) at 25 °C for 15 h. Cells were harvested by centrifugation and resuspended in Tris-HCl (30 ml of a 100 mM solution, pH 8.0) containing NaCl (150 mM) before cell lysis by sonication. The lysate was centrifuged (30,000*g*, 1 h, 4 °C) and the extract was directly loaded onto Strep-Tactin Superflow resin (Iba Life) that was previously equilibrated with Tris-HCl (100 mM, pH 8.0, containing 150 mM NaCl). Washing steps were performed using Tris-HCl (100 mM, pH 8.0), NaCl (150 mM) and Strep-tagged proteins were eluted in Tris-HCl buffer (100 mM, pH 8.0, containing 150 mM NaCl and 2.5 mM d-desthiobiotin (d-biotin)). Columns were regenerated using Tris-HCl buffer (100 mM, pH 8.0; containing 150 mM NaCl, 1 mM EDTA and 1 mM 2-(4-hydroxyphenyl-azo)benzoic acid). Eluted proteins were concentrated to a final volume of 2 ml and further purified by gel filtration (Superdex 200, in 20 mM Tris-HCl pH 8.0).

Mutant P91 C118 was purified in the same way as wild-type P91, but mutants P91 D167N and H199A were prepared using Strep-Tactin spin columns (Iba Life). Mutant-encoding plasmids were transformed by electroporation into *E. coli* BL21(DE3). Small volume cultures (15 ml) were grown under the same conditions as described above. Cells were pelleted and lysed using 500 μl of a solution containing 1 × Bugbuster (Novagen) and 0.001 × Lysonase (Novagen) in MilliQ water (Millipore) before addition of Tris-HCl buffer (1 ml, 100 mM, pH 8.0; containing 150 mM NaCl) and centrifugation to remove cell debris. After equilibration using Tris-HCl buffer, Strep-Tactin columns were loaded with the cleared cell lysates and centrifuged for 30 s at 700 g (at 4 °C). After a washing step to remove weakly bound proteins, P91 mutants were eluted in Tris-HCl (150 μl of 100 mM, pH 8.0), containing 150 mM NaCl and 2 mM d-biotin. To remove d-biotin, the elutions were buffer exchanged by successive concentration–dilution cycles using 1 ml Amicon 10 k concentrator columns.

### Substrates

Sulfate monoester **1d**, phosphotriester **2d**, phosphate diester **4** and phosphonate **5** were synthetized from the respective chlorides or chloridates^62^,^63^,^64^. Sulfate monoester **1c** was purchased from Fluka. Sulfate monoester **1a**, phosphate monoester **3**, acetate ester **6**, thioester **7**, lactones **8a**, **8b**, **8c**, **8d**_**1**_, **8d**_**2**_ and acetamide **9** were purchased from Sigma-Aldrich. Chromogenic β-lactamase substrate **10** (CENTA) was purchased from Merck Millipore.

### Enzyme Assays

All enzymatic assays were conducted at 25 °C in a final volume of 200 μl, in the activity buffer (100 mM MOPS-NaOH pH 8.0, containing 150 mM NaCl) that was used throughout the experiments, unless otherwise stated. Measurements were performed in 96-well plate format and product formation followed in SpectraMax M5 or 190 microplate readers. Hydrolysis of substrates with fluorescein leaving groups (**1d** and **2d**) was monitored at the following wavelengths: λ_excitation_=488 nm and λ_emission_=525 nm. A calibration curve (linearly fit to the equation: fluorescence=1.2 × 10^10^ M^−1^ [fluorescein]) was used to quantify fluorescein release. Hydrolysis of esters **1**, **2a**, **2b**, **3**, **4**, **5**, **6** and acetamide **9** was monitored by measuring absorbance of p-nitrophenolate at 400 nm (*ɛ*≈17,700 M^−1^ cm^−1^). Hydrolysis of thioester **7** was measured in the presence of the indicator 5,5'-dithiobis-(2-nitrobenzoic acid) (500 μM); the increase of 2-nitro-5-thiobenzoate was monitored at 412 nm (*ɛ*≈13,100 M^−1^ cm^−1^)^39^. Hydrolysis of lactones **8b**, **8c**, **8d**_**1**_, **8d**_**2**_ was measured using a pH-shift assay in 2.5 mM Bicine, 200 mM NaCl, 0.3 mM cresol purple pH 8.3 (ref. 65). The pH drop was monitored by the decrease in the absorbance of the indicator dye cresol purple at 577 nm (*ɛ*≈4,000 M^−1^ cm^−1^). Hydrolysis of lactone **8a** was measured at 270 nm in an ultraviolet-transparent 96-well plate. Hydrolysis of β-lactam **10** was measured at 405 nm in phosphate buffer (100 mM, pH 8.0). All kinetic parameters were calculated by fitting initial rates *v*_*o*_ to the Michaelis–Menten equations (or, if saturation could not be reached due to solubility limits, to linear correlations) using Kaleidagraph. Example data and details of equations used for fitting are shown in Supplementary Fig. 14.

### Sequence similarity networks

All protein sequences from superfamilies (AH: Pfam CL0034; MBLs: Pfam PF00753; α/β: Pfam PF07859, PF00135 and PF01738) were retrieved from the EMBL-EBI Pfam database^36^. To limit the number of sequences and to reduce eventual bias that could induce too many homologous sequences in a network, a cutoff on the sequence identity was applied using CD-HIT^66^ (40% for AH and MBL, and 30% for α/β). Our metagenomic hits protein sequences and several known characterized members of each protein families were subsequently added to the protein lists to help network functional annotations. The data sets were composed of 5,042 (AH), 2,984 (MBL) and 1,345 (α/β) sequences. Each data set was subjected to an all-versus-all BLAST (National Center for Biotechnology Information, version 2.2.29+), considering sequence similarity only when alignment score was below an appropriate threshold (E-values: 1 × e^−9^ (AH), 1 × e^−14^ (MBL) and 1 × e^−19^ (α/β)). The sequence similarity scores (*E*-values) were then imported into Cytoscape (version 3.0.2) and networks were constructed using the organic layout (in which the lengths of the edges correlate with the dissimilarity of the connected sequences (represented by nodes)). Functional annotations were then imported from the Uniprot database and mapped onto the networks. Recently, the process of generating SSNs from any Pfam families has been made much more convenient by the Enzyme Function Initiative: (http://efi.igb.illinois.edu/efi-est/)^67^.

### Metal removal

P83, P85, P86, P87, P88.1, P88.2, P90 and P91 were incubated at 4 °C in MOPS (100 mM, pH 8) containing NaCl (150 mM) and the chelators EDTA, (25 mM), pyridine-2,6-dicarboxylic acid (25 mM) and phenanthroline (2 mM). After 4 days of incubation, chelating agents were removed by successive centrifugations using centrifugation filter tubes (Amicon Ultra-2 ml, 10 kDa; Millipore) and washed with MOPS buffer (as above). To restore divalent metals, the samples were incubated at 25 °C with MnCl_2_ (200 μM) and activity towards phosphate triester **2a** (800 μM) was measured by monitoring the increase of 4-nitrophenolate absorbance at 400 nm. Samples that were subjected to the metal removal procedure, without restoring the metal, were used as controls to assay any residual activity in the *apo*-enzyme.

### Crystallization and structure determination

Crystallization conditions were screened in 96-well plates using the sitting-drop vapour diffusion method at 292 K. Crystals of P91 appeared after 36 h in a condition of 0.2 μl P91 (10 mg ml^−1^) and 0.2 μl reservoir solution (0.1 M Tris pH 8.5, 0.2 M MgCl_2_ and 20% (w/v) PEG 8,000). Diffraction data were collected from a single crystal at 100 K at the Swiss Light Source (beamline X06DA) at 0.9188 Å. Data were processed using autoPROC/XDS/AIMLESS software^68^. As the crystal suffered significant radiation damage, affected images were removed from processing, leading to a slightly reduced completeness of 87%—the observed electron density however is of good quality and continuous. Phases were obtained through molecular replacement using BALBES/Phaser^69^, which used a putative DLH from *Klebsiella pneumoniae* (PDB ID 3F67) as the search model. Two molecules of P91 were found in the asymmetric unit. Iterative structure refinement was performed using Refmac5 (ref. 70) from the CCP4 suite^71^ and Coot^72^. 97.7% of residues are found within the Ramachandran-favoured, 2.3% in the allowed and none in the disallowed regions. Loop 74–80 (in both monomers) shows higher flexibility than the rest of the protein and multiple conformations that could not be modelled accurately. Full data collection and refinement statistics are shown in Supplementary Table 8. The structure has been deposited with PDB code 4ZI5.

### Site-directed mutagenesis

P91 nucleophile mutant C118A was constructed by overlap extension PCR and cloned into the pASKIBA5plus (Iba Life) using the BsaI restriction site. P91 mutants D167N and H199A were constructed using the QuikChange protocol (Stratagene) with pASKIBA5plus-P91 as DNA template.

## Additional information

**Accession codes**: The sequences for all hits were submitted to the NCBI GenBank and can be found with the following accession numbers: KP212134 (*p32*), KP212135 (*p35*), KP212136 (*p40*), KP212137 (*bk1*), KP212138 (*p76*), KP212139 (*p82*), KP212140 (*p83*), KP212141 (*p84*), KP212142 (*p85*), KP212143 (*p86*), KP212144 (*p87*), KP212145 (*p88.1*), KP212146 (*p88.2*), KP212147 (*p90*), KP212148 (*p91*). The structure of P91 is available from the Protein Data Bank (ID: 4ZI5).

**How to cite this article:** Colin, P.-Y. *et al.* Ultrahigh-throughput discovery of promiscuous enzymes by picodroplet functional metagenomics. *Nat. Commun.* 6:10008 doi: 10.1038/ncomms10008 (2015).

## Supplementary Material

Supplementary Information Supplementary Figures 1-18, Supplementary Tables 1-8, Supplementary Note 1 and Supplementary References

Supplementary Movie 1 Reinjection of droplets (2 pL) into the sorting chip after an incubation of two days off-chip.

Supplementary Movie 2 Sorting of droplets (2 pL) for laser-induced fluorescence (see the fluorescence distribution in Figure 2b). The sorting rate was approximately 2 kHz.

## Figures and Tables

**Table 1:** Catalytic efficiencies of the metagenomic hits towards the tested substrates.

	**Sulfate monoester**	**Phosphate triester**		**Phosphate monoester**	**Phosphate diester**	**Phosphonate monoester**	**Ester**	**Thioester**	Lactone*
	**1a**	**2a**	**2d**	**3**	**4**	**5**	**6**	**7**	**8**
					*k*_cat_/*K*_M_ (s^−1^ M^−1^)				
P35	2 × 10^5^	—†	ND	2 × 10^−1^	9 × 10^−3^	3 × 10^1^	—	ND	ND
P83	—	3 × 10^3^	9 × 10^5^	—	—	—	1 × 10^2^	ND	5 × 10^4^
P84	—	7.6	52	—	4 × 10^−2^	3 × 10^−1^	5 × 10^2^	2 × 10^3^	2 × 10^3^
P85	—	9 × 10^1^	9 × 10^2^	—	5 × 10^−3^	2 × 10^−1^	4 × 10^1^	ND	—
P86	—	2 × 10^2^	1 × 10^3^	—	9 × 10^−3^	1 × 10^−1^	3 × 10^1^	ND	—
P87	—	1 × 10^2^	4 × 10^2^‡	—	7 × 10^−2^	1 × 10^−1^	4 × 10^1^	ND	—
P88.1	—	4 × 10^2^	3 × 10^3^	—	—	—	6 × 10^2^	ND	—
P88.2	—	2 × 10^2^	1 × 10^3^	—	—	1 × 10^−1^	1 × 10^1^	ND	—
P90	—	5 × 10^1^	2 × 10^2^	—	—	8 × 10^−1^	1 × 10^2^	2 × 10^3^	6 × 10^3^
P91	—	3 × 10^2^	9 × 10^3^	—	—	1 × 10^−1^	1 × 10^4^	ND	4 × 10^3^

ND, not determined.

The substrates tested were suggested by previous observations of promiscuity^56^ and the superfamily context (Fig. 4). Michaelis–Menten plots and all *k*_cat_ and *K*_M_ values are shown in Supplementary Fig. 14 and Supplementary Table 3, respectively.

^*^Activity measured using different lactones: **8a** (P84, P90 and P91); **8c** (P83)—no activity detected for **8b** and **8d**_1–2_ (see different lactones; Supplementary Fig. 12).

^†^(−) No activity was detected with high enzyme (1 μM) and substrate (1 mM) concentrations after incubation for 3 h.

^‡^The magnitude of the fluorescence signal change suggests that only one of the two phosphate groups of triester **2d** was cleaved.
