# Supplementary material for: Ultrahigh-throughput discovery of promiscuous enzymes by picodroplet functional metagenomics
Source: Nat Commun. 2015 Dec 7;6:10008. doi: 10.1038/ncomms10008 (PMC4686663; doi:10.1038/ncomms10008)
Supplement: Supplementary Information — Supplementary Figures 1-18, Supplementary Tables 1-8, Supplementary Note 1 and Supplementary References [file ncomms10008-s1.pdf]

Supplementary Figures

Metagenomic selections pose additional challenges compared to library selections in directed evolution. The expression of protein is generally lower, because of heterologous expression of foreign sequences in *E. coli*[1] that are randomly cloned, so that less product is typically produced. It was therefore necessary to increase the dynamic range of droplet screening to ensure hits with low activities are selected. Previous miniaturised cell lysate assay were conducted in 20 pL droplets[2]. The droplet volume was decreased by an order of magnitude to 2 pL in this work. Decreasing the droplet size should minimise the dilution of cell lysate, resulting in increased protein concentration. To demonstrate this technical facility, single cells transformed with metagenomic hit PC35 (Figure 3) were compartmentalised in droplets with 2 and 8 pL volumes (corresponding to droplet diameters of 15 vs 25 µm) and fluorescence was monitored over time using a sorting chip (Supplementary Fig. 5).

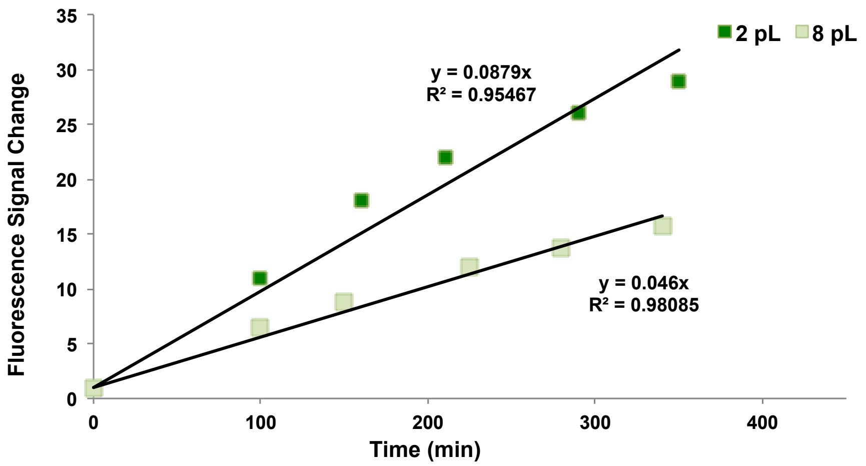

Supplementary Figure 1: Increased sensitivity in smaller droplets.
Fluorescent product was generated over time by hydrolysis of sulfate monoester **1d** by the sulfatase P35, released upon droplet encapsulation and lysis of cells transformed by the plasmid PC35. In 2 pL droplets, the occupancy (cells/droplets) was set to 0.09, whereas the occupancy was 0.04 in 8 pL droplets. The average fluorescence of occupied droplets ($\bar{F}$) was calculated using Equation 1 and normalised by the fluorescence of the majority of droplets that contain no cells to give the fluorescence signal change (FSC) (Equation 2).
Average Fluorescence ($\bar{F}$) for positive droplets (considering only droplets with fluorescence 5-times higher than the majority of droplets with low fluorescence, i.e. empty droplets).

Equation 1:

$$\bar{\mathrm{F}} = \frac{\sum(\text{Fluorescence} \times \text{Number of droplets})}{\text{Number of droplets}}$$

Fluorescence Signal Change (FSC):

Equation 2:

$$\mathrm{FSC} = \frac{\bar{\mathrm{F}}}{\text{Fluorescence of droplets without cells}}$$

a

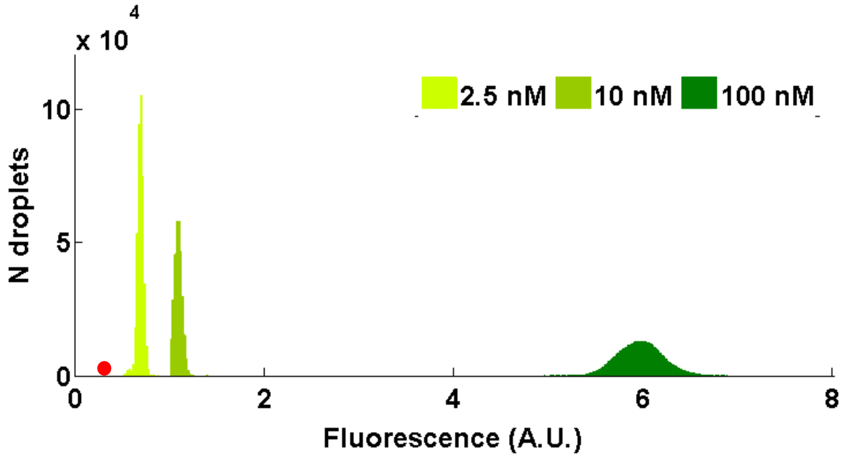

b

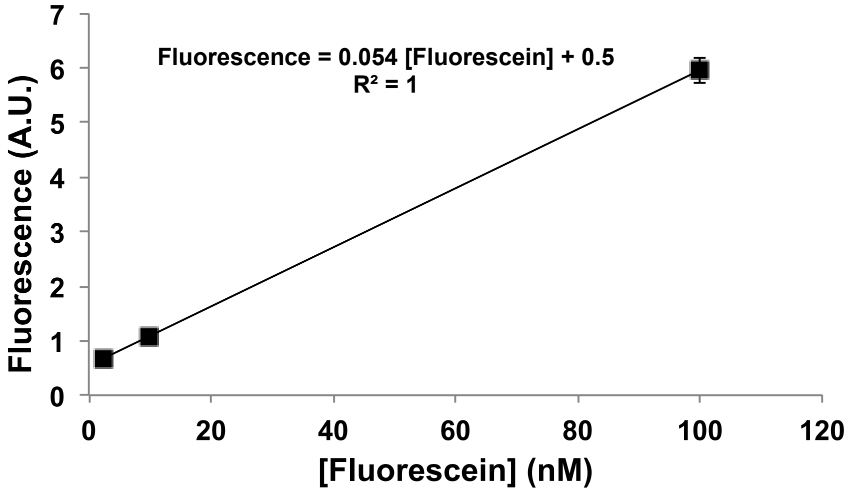

Supplementary Figure 2: Determination of the fluorescence detection limit in 2 pL droplets.

(**a**) The fluorescence signal of 2 pL droplets was measured for a range of fluorescein concentrations. A concentration as small as 2.5 nM fluorescein could be detected, corresponding to ~ 2500 fluorescein molecules in 2 pL droplets. Zero counts (within electronic noise) were observed when fluorescein concentration was below 2 nM (shown as a red dot). (**b**) Linear correlation between mean fluorescence of each measured fluorescein concentration in droplets (each measurement involved >30,000 droplets). Standard deviations of the normal distribution are reported as error bars.

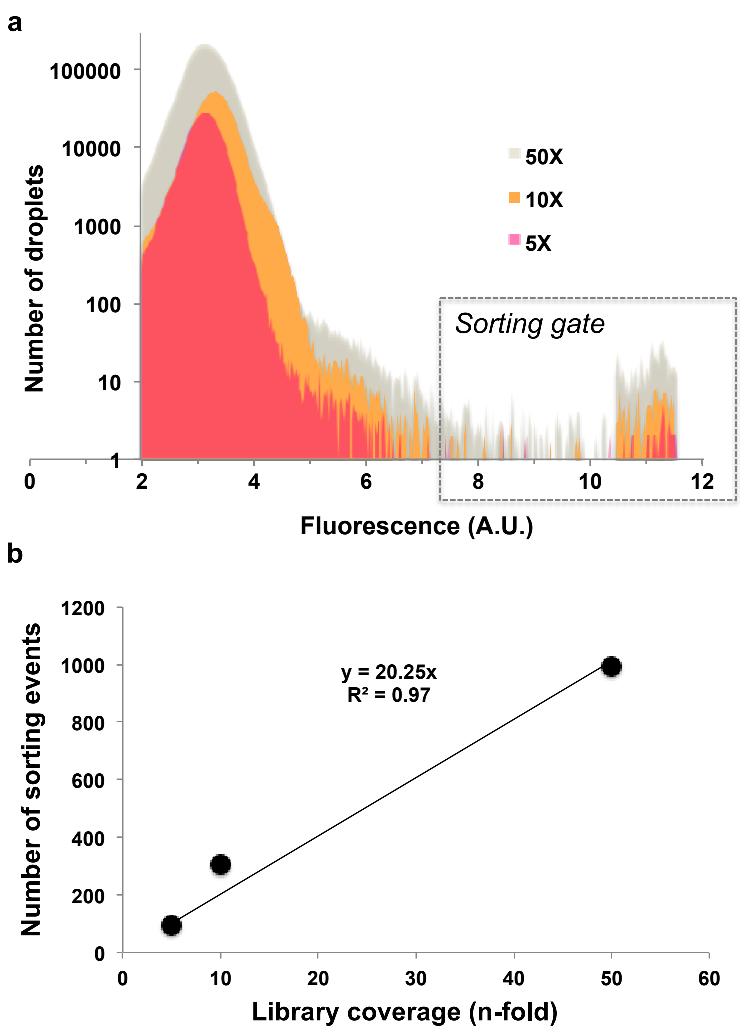

Supplementary Figure 3: Correlation between oversampling and hit rate suggests full coverage of the library content in droplet selections.

To test the reproducibility of our droplet sorter, the same library ENR-MSGL (~100,000 variants; Supplementary Table 1) was screened with three different levels of library oversampling, namely 5-fold (5X), 10-fold (10X) or 50-fold (50X).

(**a**) Overlay of histograms showing the distribution of droplets containing on average 0.8 bacteria for three different levels of library oversampling. (**b**) The linear correlation between the number of droplets in the sorting gate and the level of oversampling (or library coverage) of the library is evidence that all hits are identified under all scenarios and that with increasing oversampling the same hits are found multiple times.

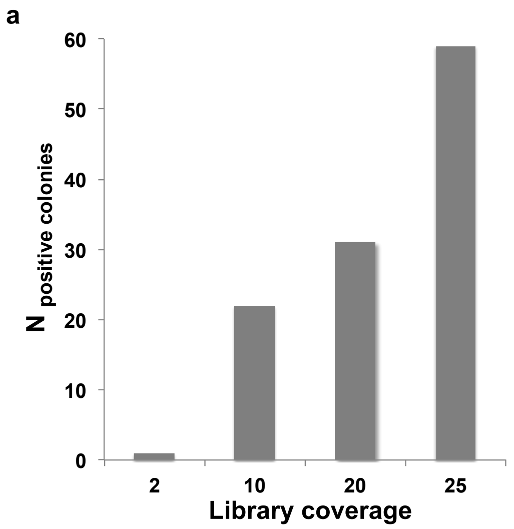

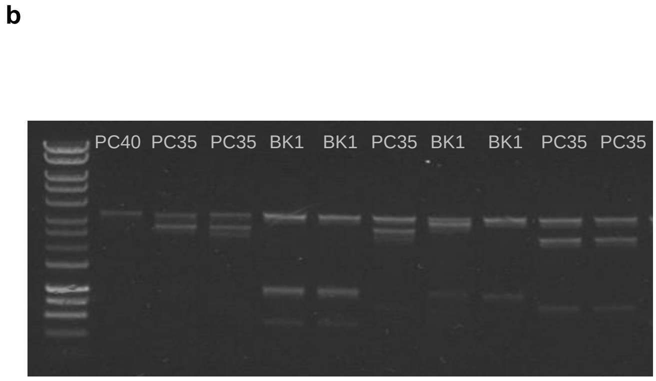

Library coverage	2	10	20	25
Unique variant	1	3	3	3

Supplementary Figure 4: Correlation between library oversampling and number of hits recovered.

To test the hypothesis that the increased screening capacity leads to a larger number of hits, a model library (containing a combination of ENR-S, ENR-G, ENR-M, ENR-L; library ENR-MSGL see Supplementary Table 1) was screened (using substrate **1d**) with oversampling of 2-, 10-, 20- and 25-fold of the theoretical library size. If the hypothesis holds, the more we oversample, the more hits we expect. To measure the outcome of selections under various coverage conditions DNA from the selected droplets was recovered and directly transformed into *E. coli*. After 2 days of growth on agar plates, transformed colonies were lysed then overlaid with a solution containing sulfate monoester **1b** (Supplementary Fig. 12). Colonies producing blue dye were identified as expressing a sulfatase. The number of positive colonies was determined and plotted against the oversampling (library coverage) (**a**). Plasmids from positive colonies were isolated and digested using BamHI and NotI. Digested plasmids were run on agarose gels and the occurrences of unique digestion patterns were determined to assess the number of unique variant of the hits (**b**). The agarose gel shows the digestion patterns for the hits PC40, PC35 and BK1. Identical digestion patterns corresponding to the three hits were found from the sorted droplets when covering the library at least 10-times. The observation of an increase in hits with larger diversity supports our hypothesis that more screening gives more hits. This experiment establishes that the throughput of the screening and selection technology is directly affecting the success of a metagenomic campaign. Ladder: Hyperladder I (Bioline).

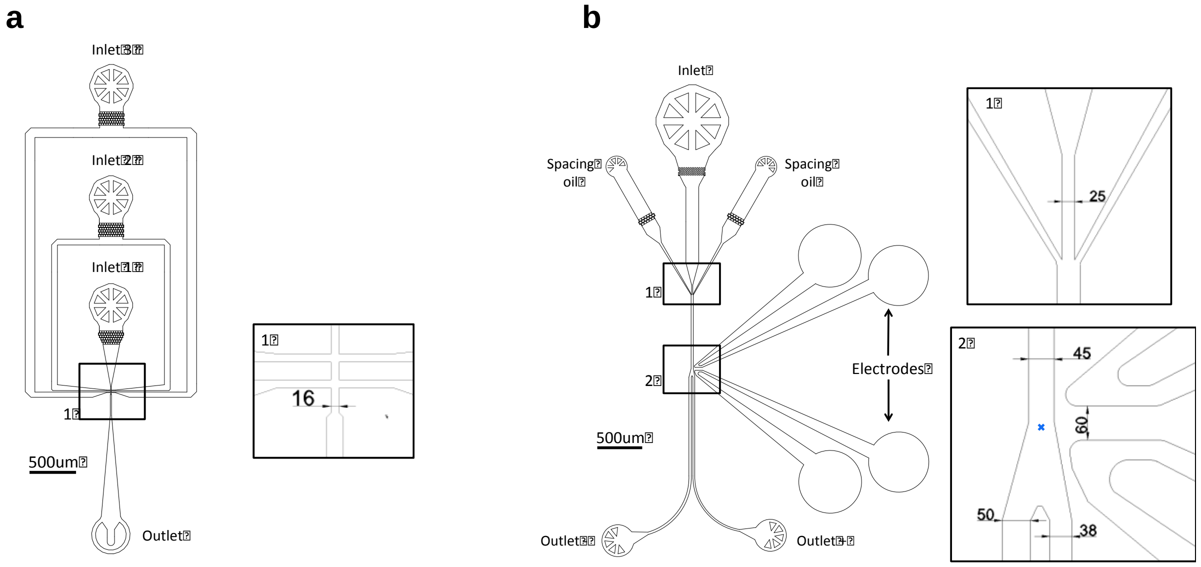

Supplementary Figure 5: Microfluidic devices used in this study.
(**a**) Flow-focusing device. Substrate and lysis agents (inlet 2) are mixed with the bacterial suspension (inlet 1) before being emulsified by fluorous oil (inlet 3); (**b**) Parallel sorting device. Droplets are re-injected and spaced out with extra fluorous oil (1). Then, droplets are selected based on their fluorescence and directed into the positives bin triggered by an electric pulse that changes the course of the droplet by dielectrophoresis. The dielectrophoretic force is related to the droplet size[3]. The absence of angle between the negative and positive channels (compared to sorting device used in Kintses *et al.*[2]) decreases the distance a deflected droplet has to travel to be selected into the positive channel making the selection of smaller droplets easier. The dimensions denoted in the drawings are given in µm. The blue cross designates the position of the laser. CAD files of the flow focusing and parallel sorter designs can be downloaded from http://www2.bio.cam.ac.uk/~fhlab/dropbase/.

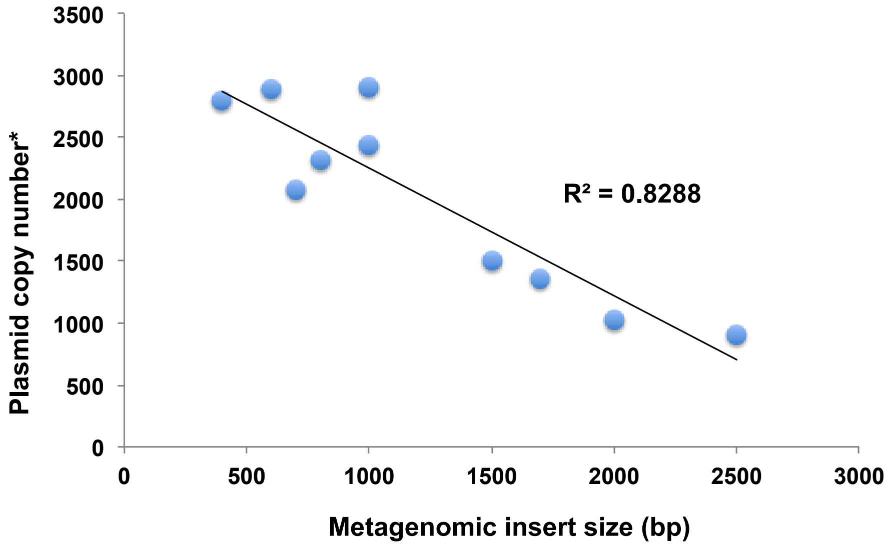

Supplementary Figure 6: Inverse correlation between insert sizes and pZERO-2 copy number.

Using PCR amplifications (on ten random metagenomic variants from libraries 1-5 and 8-10, Supplementary table 1) as insert size values the total plasmid sizes were calculated and compared to the values of copy numbers measured after DNA extraction (Miniprep, QIAGEN). *The plasmid copy numbers should not be considered as absolute values; according to manufacturer pZERO-2 copy number is ~700.

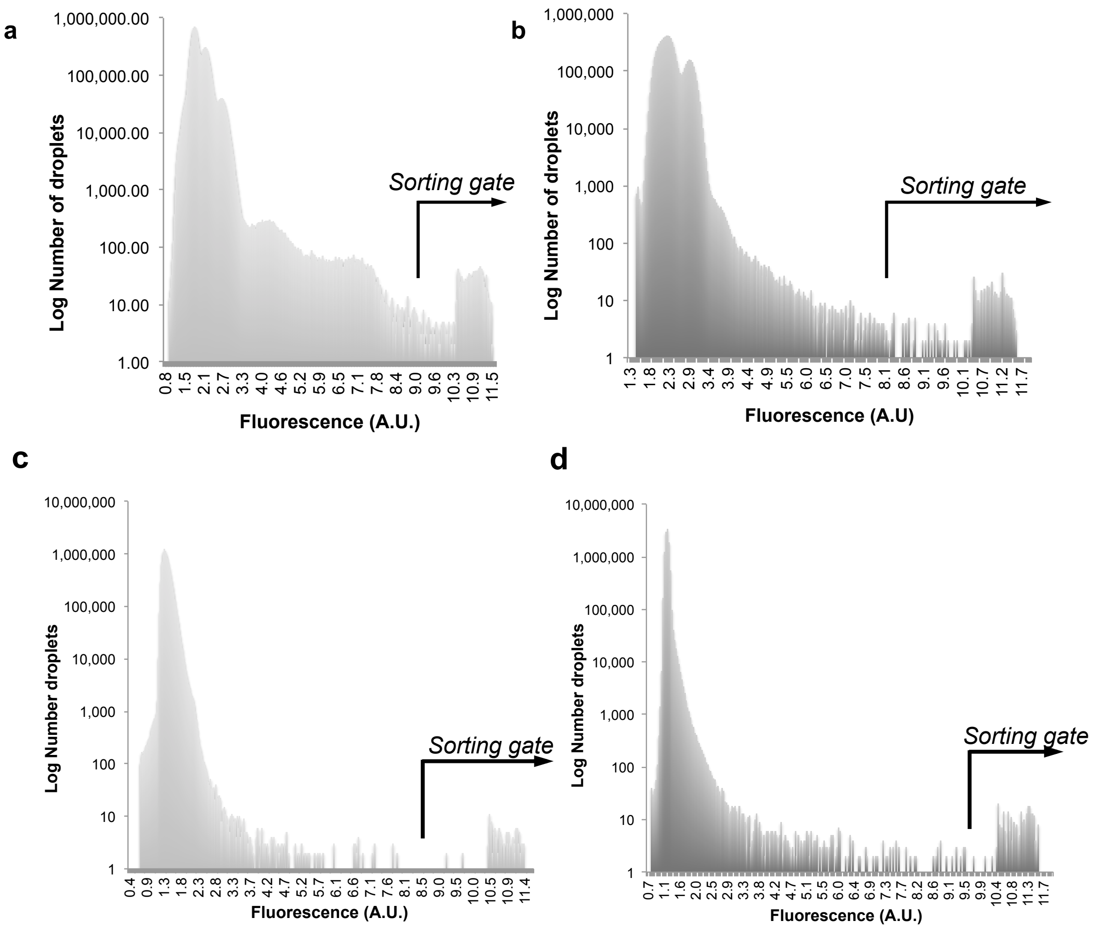

Supplementary Figure 7: Histograms of droplet fluorescence distribution for multiple screenings for sulfatases and phosphotriesterases (expanding on the data shown in Figure 2b).

These diagrams show the primary data from droplet screening along with the gate chosen for sorting. (**a**) Round 1 sulfatase screening showing a histogram representing 9.7 million droplets. (**b**) Round 2 sulfatase screening showing a histogram representing 6.7 millions droplets. (**c**) Round 1 phosphotriesterase screening showing a histogram representing 9.2 millions droplets. (**d**) Round 2 phosphotriesterase screening showing a histogram representing 10.4 million droplets. The numbers on hit rates and enrichment over the screening rounds are shown in the Supplementary Fig. 8.

The sorting gates were set at a multiple of the mean background peak, ranging between 2-fold (a and Fig. 2b) and 5-fold (d).

	Screening step	Gate ratio	Occupancy	Hit rate
Microfluidics	R1 sulfatase	0.012%	0.8	0.015%
	R2 sulfatase	0.007%	0.1	0.07%
colony	R3 sulfatase	-	-	10%
Microfluidics	R1 phosphotriesterase	0.0017%	0.8	0.0021%
	R2 phosphotriesterase	0.0028%	0.1	0.028%
plate	R3 phosphotriesterase	-	-	10%

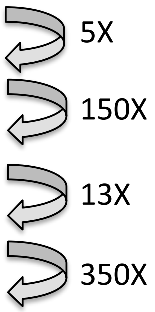

Supplementary Figure 8: Hit rates and enrichment values over multiple rounds of screening.

The hit rates were calculated by dividing the number of highly fluorescent droplets over the total number of events (gate ratio) by the occupancy. Enrichments (far right) were calculated between the different rounds. The overall enrichment approaches or exceeds three orders of magnitude (750-fold for sulfatases; 4550-fold for phosphotriesterases). When tracked at the level of *individual* genes the enrichment values can be even larger (as shown for PC86 in the main text).

a

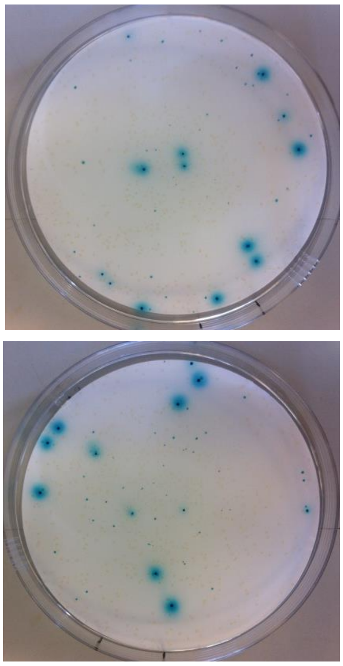

b

Plate		1	2	3	4	5	6	7	8	9	10	11	12
Plate 1	A	0.0	0.0	4.7	0.1	0.0	0.2	0.0	0.0	0.3	0.1	-0.1	0.3
	B	0.1	6.3	0.0	0.2	0.1	-0.1	0.3	0.1	0.4	0.2	0.0	9.7
	C	0.1	-0.1	0.0	0.1	0.1	0.0	0.3	0.0	0.5	0.3	3.7	0.1
	D	0.0	-0.3	0.2	9.8	0.0	-0.2	4.4	0.2	0.3	2.3	0.0	0.5
	E	6.4	-0.1	0.0	1.1	0.1	8.7	9.3	0.1	0.4	0.2	0.1	0.2
	F	0.0	0.0	0.0	0.3	0.1	0.2	9.3	0.2	0.3	0.2	5.4	0.2
	G	6.9	2.8	-0.1	0.2	0.1	0.1	1.4	0.2	0.3	0.4	0.1	0.1
	H	-0.1	-0.1	0.0	0.1	0.2	0.0	0.1	2.4	0.3	0.2	0.1	0.0
Plate 2	A	-0.5	-0.3	1.9	-0.5	0.9	1.3	0.7	-0.4	-0.5	-0.5	-0.5	-0.6
	B	-0.5	2.4	3.9	1.6	1.5	-0.5	-0.6	-0.4	-0.6	-0.4	-0.3	-0.6
	C	1.4	-0.6	1.4	-0.5	-0.5	-0.6	-0.6	-0.4	-0.4	-0.5	-0.5	-0.5
	D	-0.5	-0.7	-0.3	0.6	-0.5	-0.5	-0.6	-0.4	-0.5	-0.4	-0.5	-0.5
	E	0.9	2.5	-0.4	-0.5	-0.5	-0.5	-0.5	-0.4	-0.4	-0.5	-0.5	-0.5
	F	-0.5	-0.5	-0.4	-0.5	-0.5	-0.5	-0.5	-0.5	-0.5	-0.5	2.3	-0.5
	G	3.7	1.5	0.7	1.3	-0.5	-0.5	-0.6	-0.5	-0.4	-0.5	0.6	-0.6
	H	-0.5	-0.5	-0.4	-0.4	-0.5	-0.5	2.6	-0.5	-0.5	3.2	-0.4	-0.5
Plate 3	A	-0.1	-0.1	0.0	0.0	-0.2	-0.2	-0.1	2.5	-0.1	-0.1	0.0	0.0
	B	2.8	6.1	-0.1	-0.2	-0.2	-0.3	0.0	-0.1	-0.3	5.9	-0.3	0.0
	C	-0.2	0.0	-0.2	-0.1	5.1	-0.2	-0.2	-0.1	-0.3	-0.1	0.0	5.7
	D	0.1	-0.2	-0.3	0.1	-0.4	0.0	-0.3	0.0	-0.2	-0.2	-0.1	0.1
	E	0.1	-0.2	-0.1	7.1	3.7	-0.2	0.0	0.0	-0.2	-0.1	0.0	5.1
	F	-0.1	-0.2	0.0	0.1	-0.2	-0.1	0.0	0.0	-0.2	0.0	4.9	0.5
	G	6.0	-0.2	-0.1	0.1	-0.1	-0.1	0.1	0.0	-0.1	-0.1	0.0	0.2
	H	-0.1	-0.2	0.0	0.2	-0.1	-0.2	-0.1	0.2	-0.1	6.4	-0.1	-0.1
Plate 4	A	0.0	0.1	0.1	0.3	0.0	0.0	0.1	0.0	0.0	0.2	0.0	-0.2
	B	0.3	4.9	0.0	6.6	-0.1	0.0	0.0	6.1	7.2	0.1	-0.1	-0.1
	C	0.3	-0.1	-0.1	0.0	-0.1	-0.1	-0.1	0.0	0.0	0.0	-0.1	-0.4
	D	0.1	-0.2	4.9	0.3	-0.3	-0.1	-0.1	0.0	0.0	-0.4	0.0	-0.1
	E	0.2	0.0	6.5	0.1	0.0	-0.1	0.0	-0.1	0.0	0.0	-0.1	-0.2
	F	0.3	0.0	8.0	0.0	0.0	-0.1	-0.1	0.0	-0.1	0.0	6.4	0.2
	G	0.1	0.0	7.0	0.1	0.0	-0.1	0.0	0.0	-0.1	6.4	0.1	0.2
	H	-0.3	0.1	0.3	0.1	0.0	0.0	-0.1	6.3	0.0	0.0	0.0	-0.3

Supplementary Figure 9: Validation of the activity of hits towards sulfate monoester (a) and phosphotriester (b).

(**a**) ~1,200 transformants grew on plate after transformation of plasmids (2.5 out of 7 μL) recovered from selected droplets after the 2nd round of microfluidic screening. 104 (~10%) displayed sulfatase activity (colonies turning blue after lysis, overlay with sulfate monoester **1b** and overnight incubation at 22°C – as described in Metagenomic screening on plates) ~half of the colonies that turned blue were further confirmed as positive hits using microtiter plate (using substrate **1d**) and the positive variants were sequenced. (**b**) ~1,200 transformants grew on plates and 368 clones were randomly selected to be re-grown in liquid culture (1 mL). Following overnight growth at 37 °C and incubation for 24 hours at 22 °C, cultures were pelleted and lysed in order to assay phosphotriesterase activity towards triester **2d**. 42 variants (~10%) displayed at least a 2-fold increase (shown in green) of the fluorescence signal, when compared to a negative control. Metagenomic variants without phosphotriesterase activity were used as negative controls and are shown here in wells 2C and 11G of each plate) in a 40 hour endpoint measurement. A variant expressing a mutant of *Pseudomonas diminuta* phosphotriesterase was used as a positive control (here in wells 2B and 11F of each plate). Plasmids from positive variants were isolated and sequenced.

a. Number of total plasmid copies and plasmid PC86 per DNA sample

	Total plasmid copies / µL DNA	PC86 plasmid copies / µL DNA	Ratio
Before Selection	$3.4(\pm2.2)\ x10^{9}$	16±3	$4.6(\pm3.2)x10^{-9}$
Round 1	$2.5(\pm0.4)x10^{5}$	6±1	$2.3(\pm0.5)x10^{-5}$
Round 2	$2.7(\pm0.1)x10^{3}$	9±3	$3.5(\pm2.1)x10^{-3}$

b. Enrichment data over the 2 microfluidic rounds

	Enrichment
After Round 1	$\sim5x10^{3}$
After Round 2	$\sim10^{2}$
Total enrichment	$\sim7x10^{5}$

c. DNA recovery from droplets and transformation efficiency

	Before selection		Round 1		Round 2	
	PC86	All plasmid	PC86	All plasmid	PC86	All plasmid
N transformants	-	-	5	50000	20	1500
Transformation efficiency	-	-	100	443	100	443
N plasmids	180	40000000000	532	22150000	2000	664667
qPCR (%)	0.0000005	99.9999995	0.0024	99.9976	0.3	99.7
Plasmids/cell	~500	~2000	~500	~2000	~500	~2000
N sorted cells	1	20,000,000	1	11075	4	332
N sorted droplets	-	-	2	11075	4	300

Supplementary Figure 10: Quantification of microfluidic enrichment of the hit PC86 by qPCR.
The number of plasmids encoding the phosphotriesterase hit PC86 was measured after each rounds of selections and compared to the total number of plasmids. (**a**). Number of total plasmid copies and plasmid PC86 per DNA sample. The plasmid copy numbers were normalised by the volume of DNA used for the qPCR assays. (**b**). Enrichment data. The enrichment value after round 1 was obtained by comparing the plasmid Ratio R ((Number PC86/µL DNA)/(Total plasmids/ µL DNA)) from the table in panel (**a**) (*i.e*. Enrichment = $R^{Round\ 1}/R^{before\ selection}$) and analogously for round 2. (**c**) DNA recovery from droplets and transformation efficiency for the enrichment of a phosphotriesterase hit (PC86). Numbers indicated in red are experimentally obtained (from qPCR analysis, quantification of the number of transformants/plate, the number of sorted droplets and the plasmid copy number). Numbers in black are calculated values.

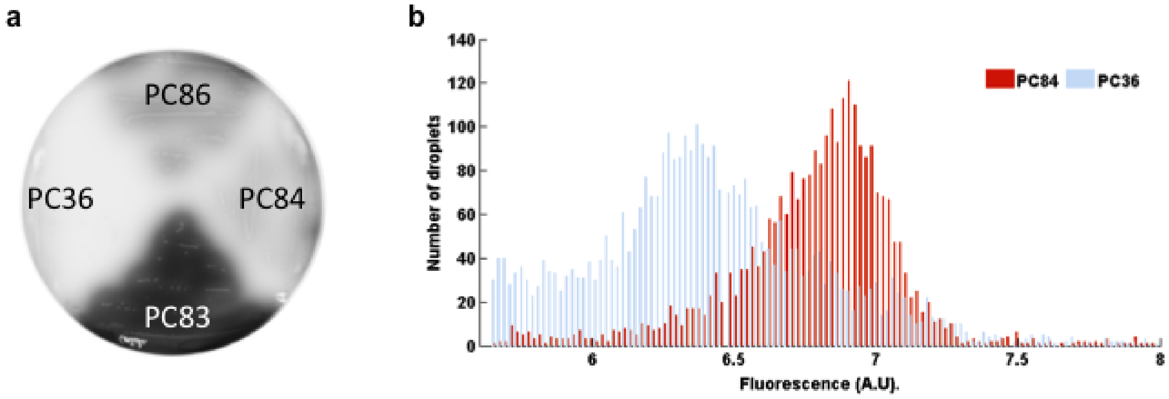

Supplementary Figure 11: Recovering weak activities from metagenomes using microdroplets.

(**a**) We measured fluorescent signals of lysed colonies of a metagenomic variant (PC36 without measurable triesterase activity; randomly picked) and three phosphotriesterase hits (PC83, PC84 and PC86 containing respectively the gene p83, p84 and p86 coding for triesterases with different catalytic efficiencies (Table 1)). This colony-screening assay for activity (using **2d** as substrate) generated a very small fluorescence signal on colonies expressing P84 (k_{cat}/K_M ~ 57 $M^{-1}s^{-1}$) even when spread as lawn (and distinction of individual PC36 and PC84 colonies was impossible) whereas higher fluorescence was observed for the hits expressing stronger phosphotriesterases (P83 and P86; k_{cat}/K_M ~$9x10^5$ $M^{-1}s^{-1}$ and ~10^3 $M^{-1}s^{-1}$ respectively). (**b**) Using the same substrate **2d** and growth/expression conditions (growth on ∅ 14 cm-plates at 37 °C for 15 h and incubated at 22 °C for 24 h), we used microfluidic droplets (as described throughout this paper) to test whether a quantitative distinction between bacteria transformed with PC84 and PC36 was possible. The fluorescence distributions for PC36 and PC84 can be clearly distinguished in the readout of droplet screening. However, the fluorescence signals of the weak phosphotriesterase of PC84 and the negative variant PC36 overlap (after 24 h). When a selection threshold of 2- to 5-fold (increase over average fluorescence of ‘negatives’, i.e. droplets not containing a hit) was chosen, this overlap explains how negative variants are still present in the selected clones. Such low selection threshold is necessary to ensure that weak catalysts such as PC84 are not missed.

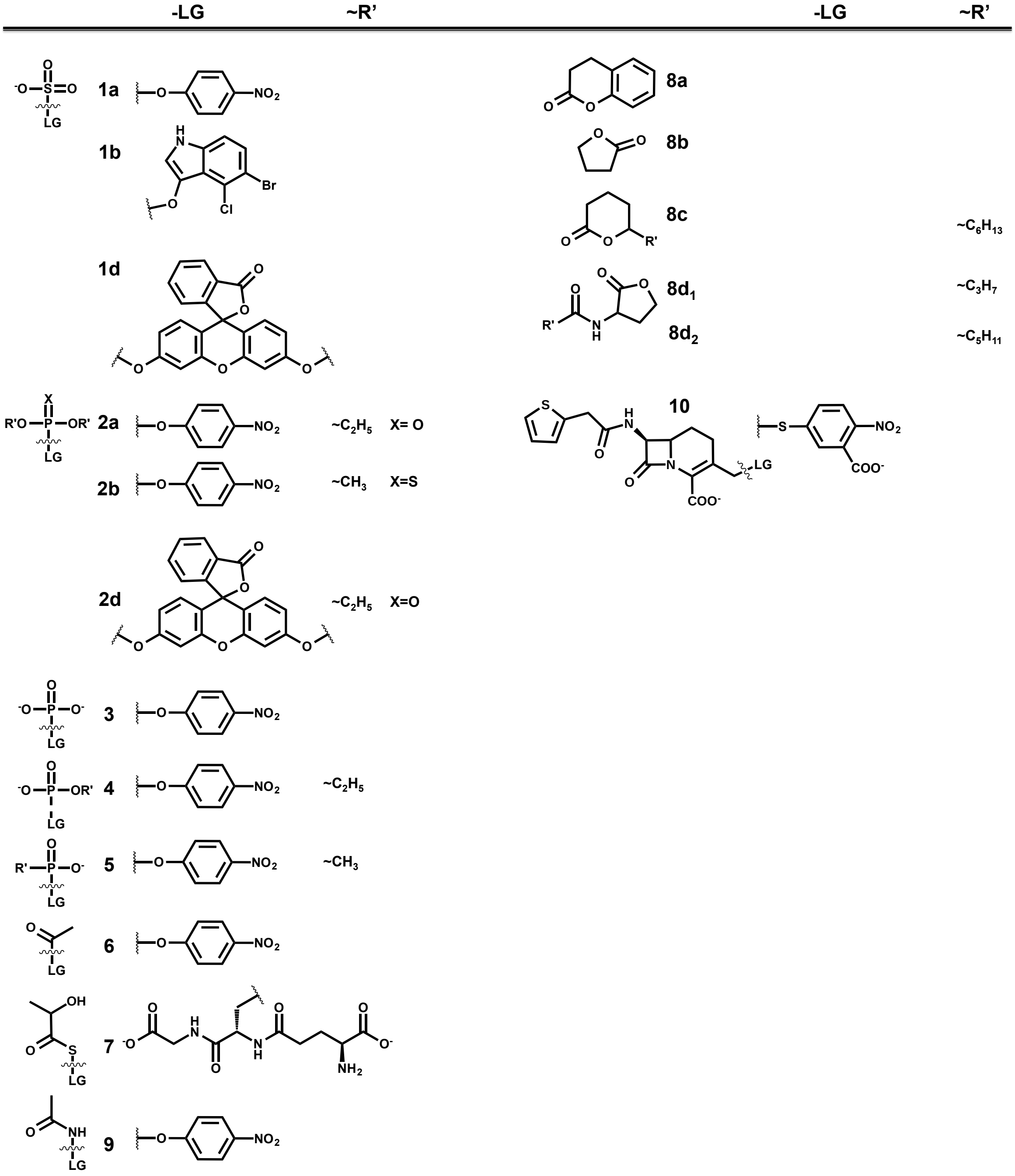

Supplementary Figure 12: Substrates used in this study.
Kinetic parameters for each substrate are presented in Supplementary Table 3. We note that no activity was found for any metagenomic hits towards lactones **8b**, **8c**, **8d$_{1\text{-}2}$**, amide **9** and β-lactam **10** under conditions described in Supplementary Fig. 14. LG: leaving group.

a

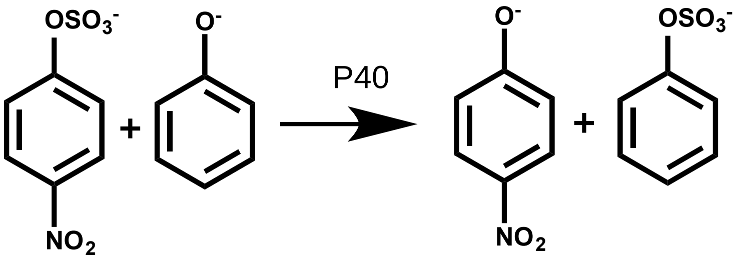

A (**1a**) B

b

k_{cat} (s^{-1})	$K_{M\ A}$ (mM)	$K_{M\ B}$ (mM)	$K_{i\ B}$ (mM)
2.6 (± 0.2)	0.07 (± 0.01)	3.0 (± 0.3)	138 (± 13)

Supplementary Figure 13: Reaction catalysed by arylsulfotransferase P40. (a) Reaction scheme. (b) kinetic parameters based on a fit of initial rates v_o to a ping-pong mechanism (Equation 3) (with A = sulfate monoester **1a** and B = phenolate).

Conditions: [HNa_2PO4/H_2NaPO_4] = 100 mM, pH 8.0 at 25 °C.

Equation 3: $$v = \frac{k_{cat}\,[A]\,[B]}{[A][B] + K_{M\,A}\,[B]\,\left(1 + \frac{[B]}{K_{i\,B}}\right) + K_{M\,B}\,[A]}$$

Supplementary Figure 14: Michaelis Menten plots for all reactions measured in this study.
Kinetics measured for sulfatase P35 (a) and phosphotriesterases P83 (b), P84 (c), P85 (d), P86 (e), P87 (f), P881 (g), P882 (h), P90 (i), P91 (j).
Kinetic parameters were obtained in Kaleidagraph by fitting initial rates to the following equations:

(*i*) the Michaelis- Menten equation $v = k_{cat} [S] / (K_M + [S])$;

(*ii*) the Michaelis-Menten with substrate inhibition:

$$v = k_{cat} [S] / (K_M + [S] + ([S]^2/K_i));$$

In cases where limited substrate solubility precluded measurement up to 10 x K_M, fits to the Michaelis-Menten equation were performed, however, in these cases the data carry a larger uncertainty than indicated by the error calculated.
For substrates with a fluorescein leaving group monophasic kinetics were observed, so it was assumed that both ester groups are cleaved with similar efficiency, except for P87 which cleaved only one ester group. The reaction conditions are as specified in Supplementary Table 3.

For substrates **2a** (~5mM), **2b** (~5mM), **3** (~60 mM**)**, **4** (~70 mM), **5** (~10 mM), **6** (~4 mM), **7** (~2 mM) and **8a** (~5 mM), limited substrate solubility at pH 8 precluded measurements up to 10 x K_M.

a. P35

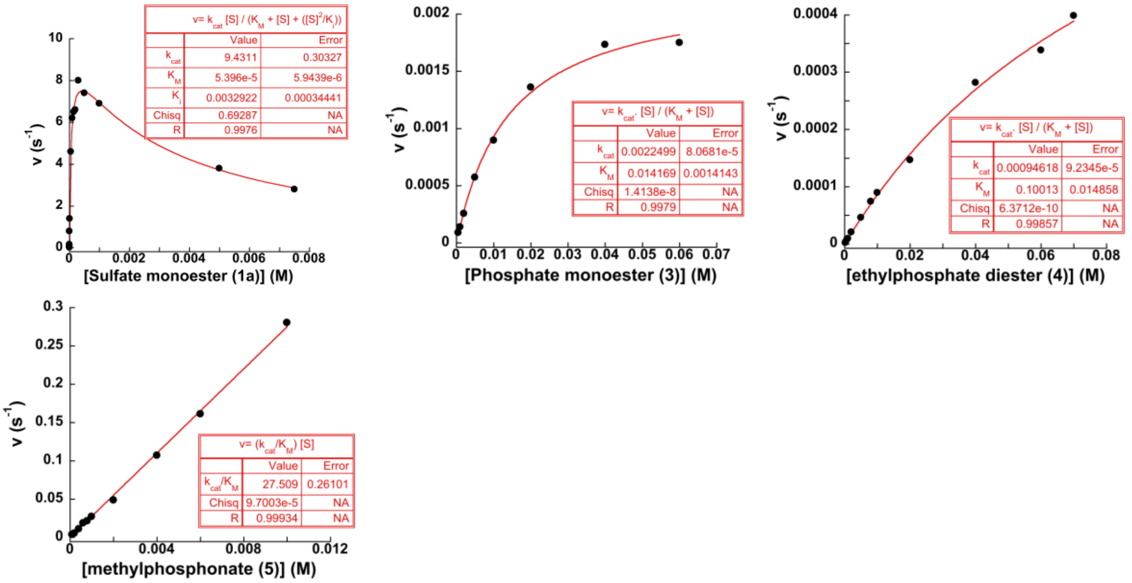

b. P83

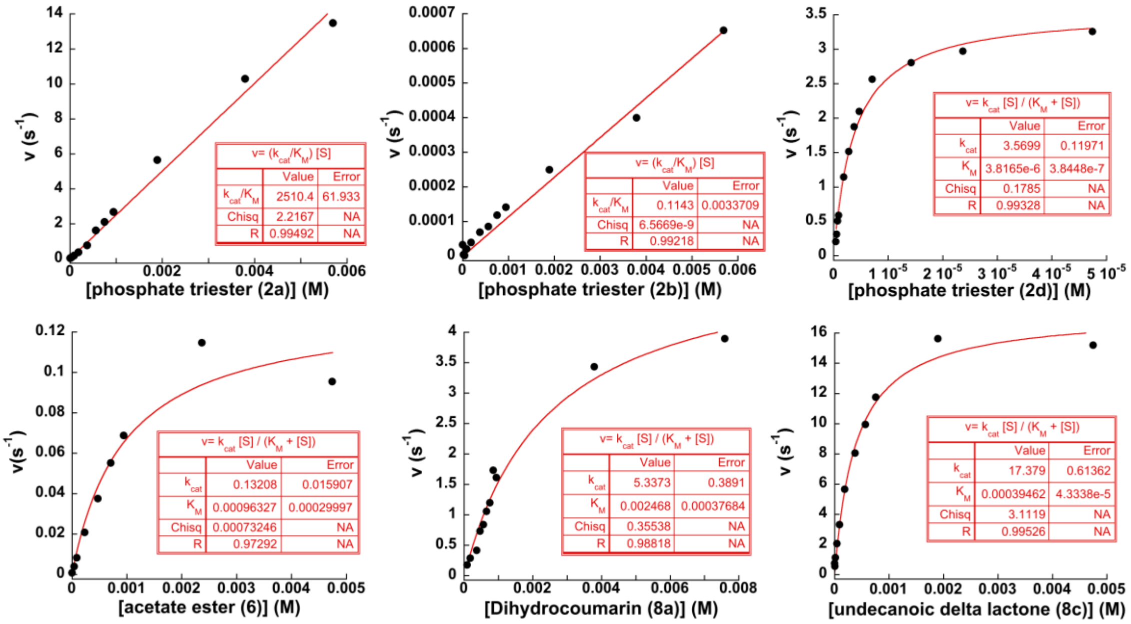
v (s-1)
[phosphate triester (2a)] (M)
v= (kcat/KM) [S]
Value Error
kcat/KM 2510.4 61.933
Chisq 2.2167 NA
R 0.99492 NA
[phosphate triester (2b)] (M)
v= (kcat/KM) [S]
Value Error
kcat/KM 0.1143 0.0033709
Chisq 6.5669e-9 NA
R 0.99218 NA
[phosphate triester (2d)] (M)
v= kcat [S] / (KM + [S])
Value Error
kcat 3.5699 0.11971
KM 3.8165e-6 3.8448e-7
Chisq 0.1785 NA
R 0.99328 NA
[acetate ester (6)] (M)
v= kcat [S] / (KM + [S])
Value Error
kcat 0.13208 0.015907
KM 0.00096327 0.00029997
Chisq 0.00073246 NA
R 0.97292 NA
[Dihydrocoumarin (8a)] (M)
v= kcat [S] / (KM + [S])
Value Error
kcat 5.3373 0.3891
KM 0.002468 0.00037684
Chisq 0.35538 NA
R 0.98818 NA
[undecanoic delta lactone (8c)] (M)
v= kcat [S] / (KM + [S])
Value Error
kcat 17.379 0.61362
KM 0.00039462 4.3338e-5
Chisq 3.1119 NA
R 0.99526 NA

c. P84

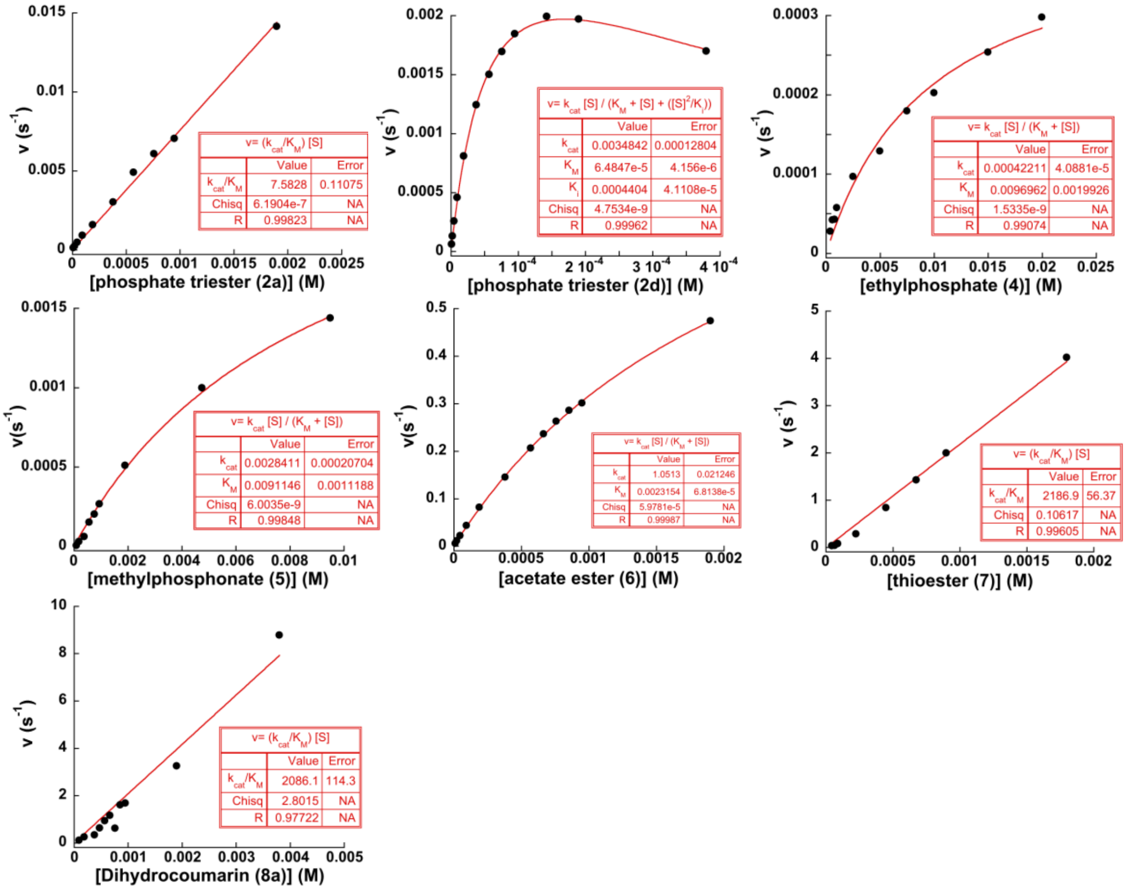
v (s-1)
[phosphate triester (2a)] (M)
v= (kcat/KM) [S]
Value Error
kcat/KM 7.5828 0.11075
Chisq 6.1904e-7 NA
R 0.99823 NA
[phosphate triester (2d)] (M)
v= kcat [S] / (KM + [S] + ([S]2/Ki))
Value Error
kcat 0.0034842 0.00012804
KM 6.4847e-5 4.156e-6
Ki 0.0004404 4.1108e-5
Chisq 4.7534e-9 NA
R 0.99962 NA
[ethylphosphate (4)] (M)
v= kcat [S] / (KM + [S])
Value Error
kcat 0.00042211 4.0881e-5
KM 0.0096962 0.0019926
Chisq 1.5335e-9 NA
R 0.99074 NA
[methylphosphonate (5)] (M)
v= kcat [S] / (KM + [S])
Value Error
kcat 0.0028411 0.00020704
KM 0.0091146 0.0011188
Chisq 6.0035e-9 NA
R 0.99848 NA
[acetate ester (6)] (M)
v= kcat [S] / (KM + [S])
Value Error
kcat 1.0513 0.021246
KM 0.0023154 6.8138e-5
Chisq 5.9781e-5 NA
R 0.99987 NA
[thioester (7)] (M)
v= (kcat/KM) [S]
Value Error
kcat/KM 2186.9 56.37
Chisq 0.10617 NA
R 0.99605 NA
[Dihydrocoumarin (8a)] (M)
v= (kcat/KM) [S]
Value Error
kcat/KM 2086.1 114.3
Chisq 2.8015 NA
R 0.97722 NA

d. P85

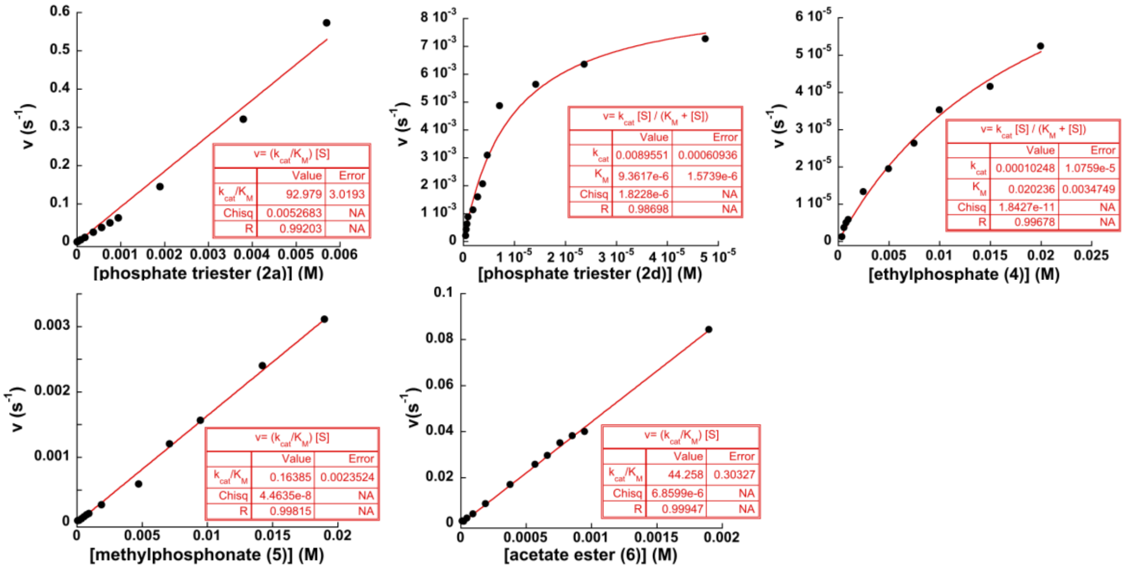

v (s-1)
[phosphate triester (2a)] (M)
v= (kcat/KM) [S]
Value Error
kcat/KM 92.979 3.0193
Chisq 0.0052683 NA
R 0.99203 NA
[phosphate triester (2d)] (M)
v= kcat [S] / (KM + [S])
Value Error
kcat 0.0089551 0.00060936
KM 9.3617e-6 1.5739e-6
Chisq 1.8228e-6 NA
R 0.98698 NA
[ethylphosphate (4)] (M)
v= kcat [S] / (KM + [S])
Value Error
kcat 0.00010248 1.0759e-5
KM 0.020236 0.0034749
Chisq 1.8427e-11 NA
R 0.99678 NA
[methylphosphonate (5)] (M)
v= (kcat/KM) [S]
Value Error
kcat/KM 0.16385 0.0023524
Chisq 4.4635e-8 NA
R 0.99815 NA
[acetate ester (6)] (M)
v= (kcat/KM) [S]
Value Error
kcat/KM 44.258 0.30327
Chisq 6.8599e-6 NA
R 0.99947 NA

e. P86

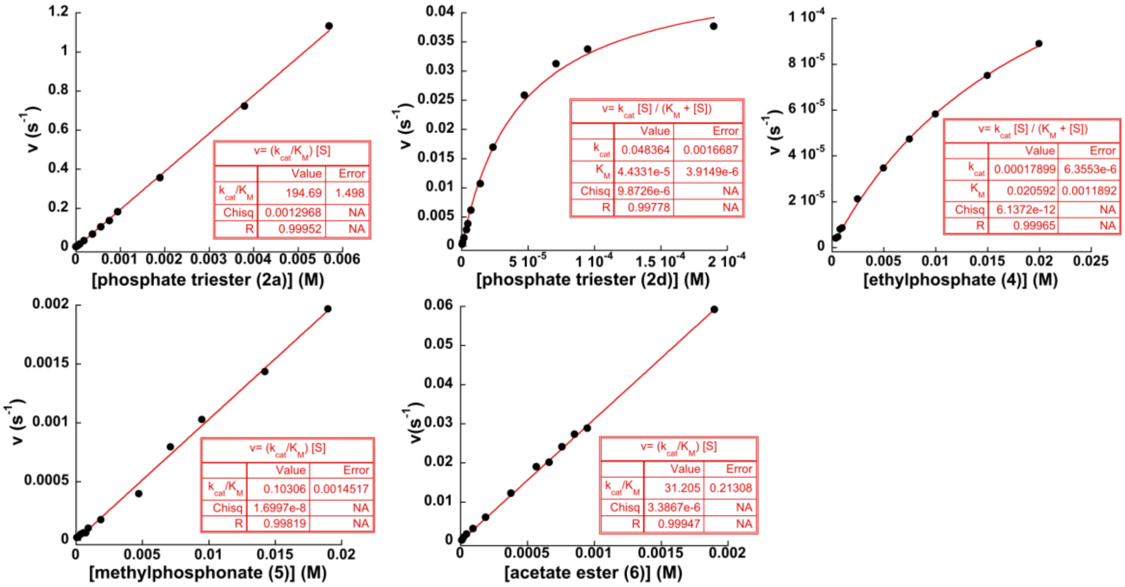

v (s-1)
[phosphate triester (2a)] (M)
v= (kcat/KM) [S]
Value Error
kcat/KM 194.69 1.498
Chisq 0.0012968 NA
R 0.99952 NA
[phosphate triester (2d)] (M)
v= kcat [S] / (KM + [S])
Value Error
kcat 0.048364 0.0016687
KM 4.4331e-5 3.9149e-6
Chisq 9.8726e-6 NA
R 0.99778 NA
[ethylphosphate (4)] (M)
v= kcat [S] / (KM + [S])
Value Error
kcat 0.00017899 6.3553e-6
KM 0.020592 0.0011892
Chisq 6.1372e-12 NA
R 0.99965 NA
[methylphosphonate (5)] (M)
v= (kcat/KM) [S]
Value Error
kcat/KM 0.10306 0.0014517
Chisq 1.6997e-8 NA
R 0.99819 NA
[acetate ester (6)] (M)
v= (kcat/KM) [S]
Value Error
kcat/KM 31.205 0.21308
Chisq 3.3867e-6 NA
R 0.99947 NA

f. P87

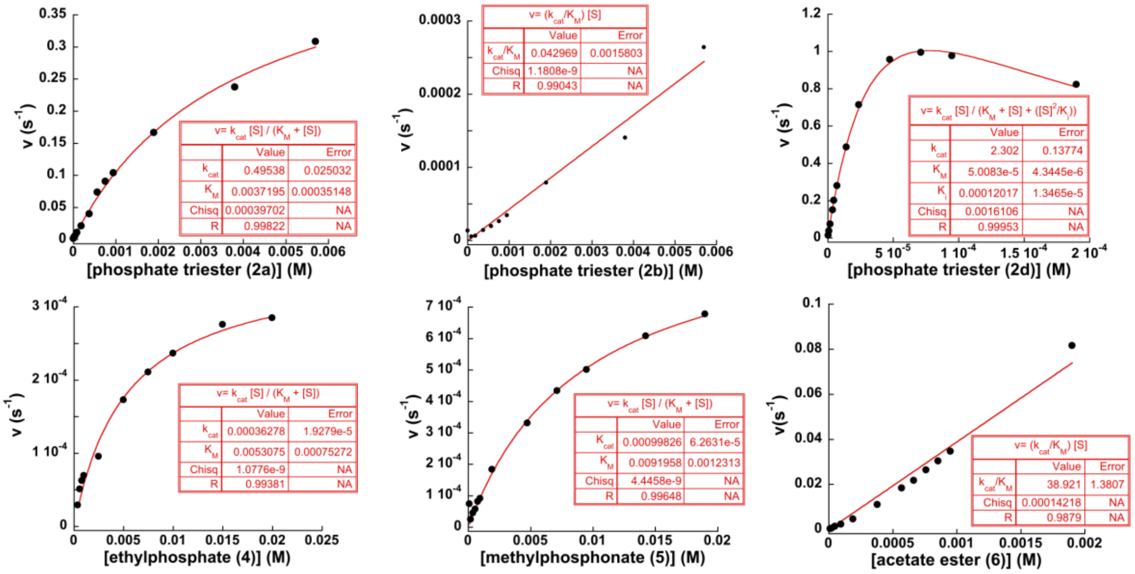

Kinetics of hydrolysis of phosphate triester **2d** were analysed using a reference curve with fluorescein-mono (diethyl phosphate).

g. P88.1

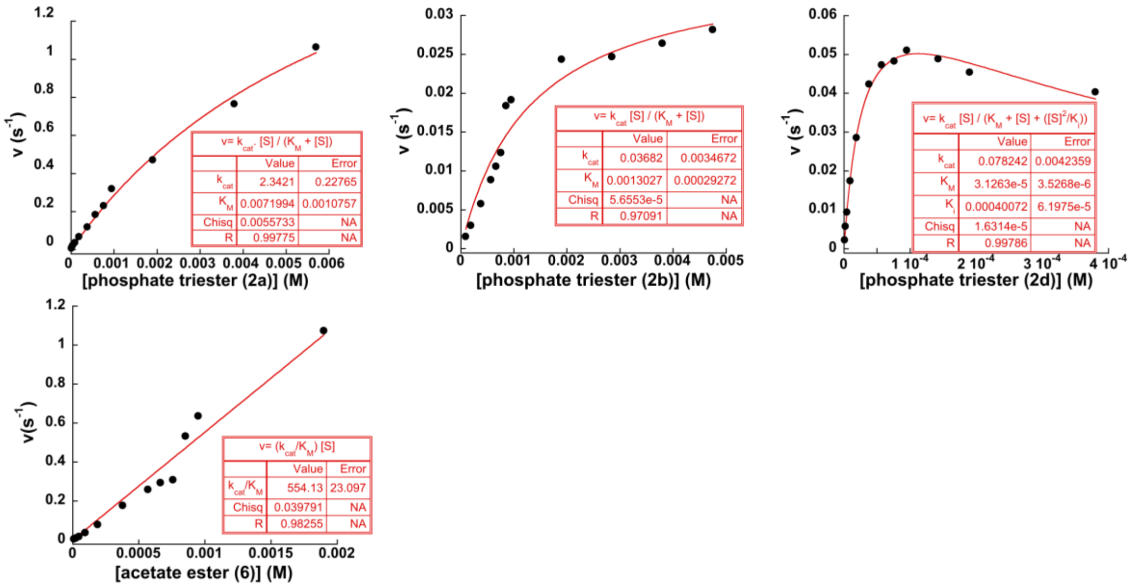

h. P88.2

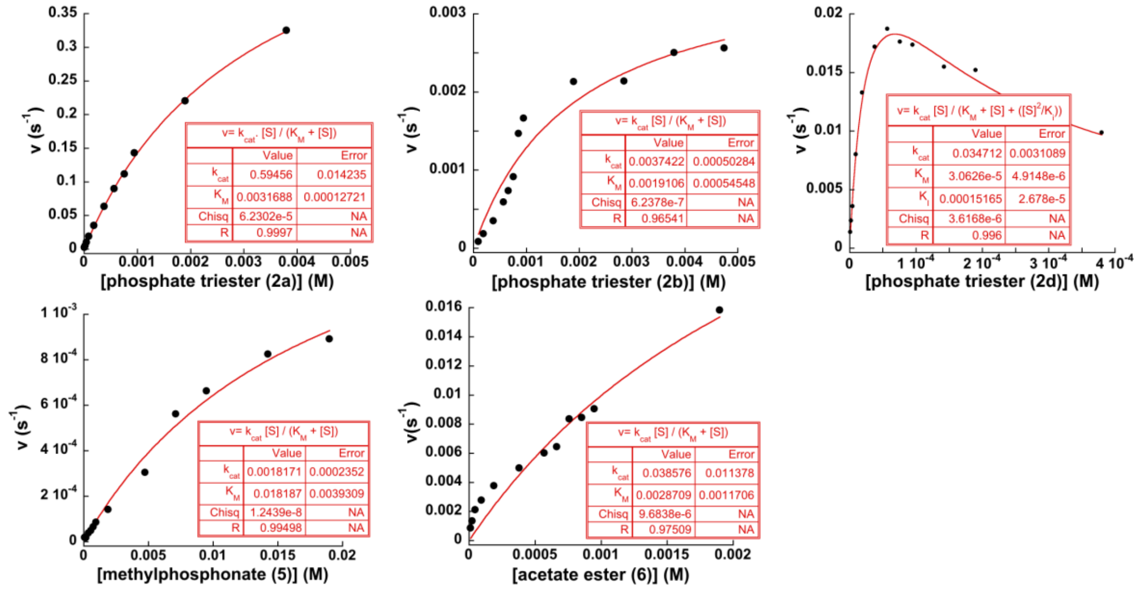
v (s-1)
[phosphate triester (2a)] (M)
v= kcat. [S] / (KM + [S])
Value Error
kcat 0.59456 0.014235
KM 0.0031688 0.00012721
Chisq 6.2302e-5 NA
R 0.9997 NA
[phosphate triester (2b)] (M)
v= kcat [S] / (KM + [S])
Value Error
kcat 0.0037422 0.00050284
KM 0.0019106 0.00054548
Chisq 6.2378e-7 NA
R 0.96541 NA
[phosphate triester (2d)] (M)
v= kcat [S] / (KM + [S] + ([S]2/Ki))
Value Error
kcat 0.034712 0.0031089
KM 3.0626e-5 4.9148e-6
Ki 0.00015165 2.678e-5
Chisq 3.6168e-6 NA
R 0.996 NA
[methylphosphonate (5)] (M)
v= kcat [S] / (KM + [S])
Value Error
kcat 0.0018171 0.0002352
KM 0.018187 0.0039309
Chisq 1.2439e-8 NA
R 0.99498 NA
[acetate ester (6)] (M)
v= kcat [S] / (KM + [S])
Value Error
kcat 0.038576 0.011378
KM 0.0028709 0.0011706
Chisq 9.6838e-6 NA
R 0.97509 NA

i. P90

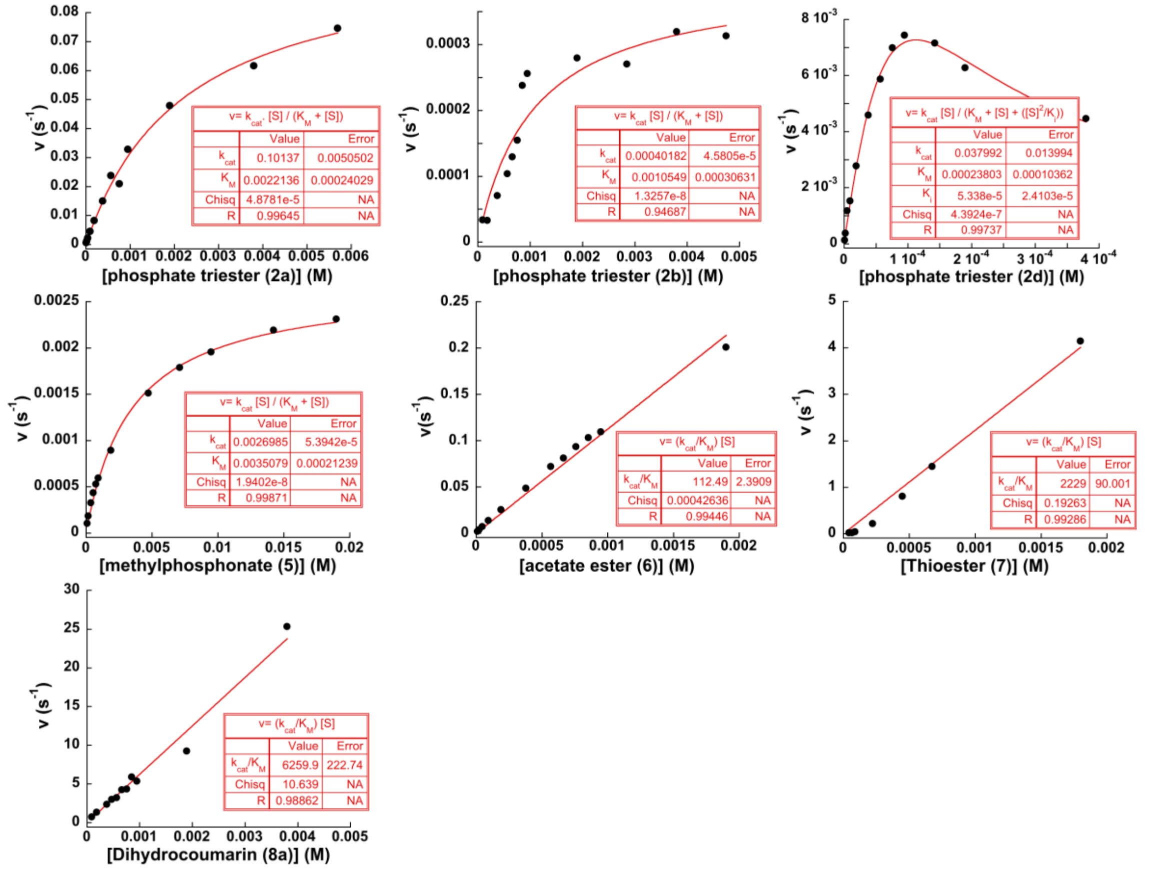
v (s-1)
[phosphate triester (2a)] (M)
v= kcat. [S] / (KM + [S])
Value Error
kcat 0.10137 0.0050502
KM 0.0022136 0.00024029
Chisq 4.8781e-5 NA
R 0.99645 NA
[phosphate triester (2b)] (M)
v= kcat [S] / (KM + [S])
Value Error
kcat 0.00040182 4.5805e-5
KM 0.0010549 0.00030631
Chisq 1.3257e-8 NA
R 0.94687 NA
[phosphate triester (2d)] (M)
v= kcat [S] / (KM + [S] + ([S]2/Ki))
Value Error
kcat 0.037992 0.013994
KM 0.00023803 0.00010362
Ki 5.338e-5 2.4103e-5
Chisq 4.3924e-7 NA
R 0.99737 NA
[methylphosphonate (5)] (M)
v= kcat [S] / (KM + [S])
Value Error
kcat 0.0026985 5.3942e-5
KM 0.0035079 0.00021239
Chisq 1.9402e-8 NA
R 0.99871 NA
[acetate ester (6)] (M)
v= (kcat/KM) [S]
Value Error
kcat/KM 112.49 2.3909
Chisq 0.00042636 NA
R 0.99446 NA
[Thioester (7)] (M)
v= (kcat/KM) [S]
Value Error
kcat/KM 2229 90.001
Chisq 0.19263 NA
R 0.99286 NA
[Dihydrocoumarin (8a)] (M)
v= (kcat/KM) [S]
Value Error
kcat/KM 6259.9 222.74
Chisq 10.639 NA
R 0.98862 NA

j. P91

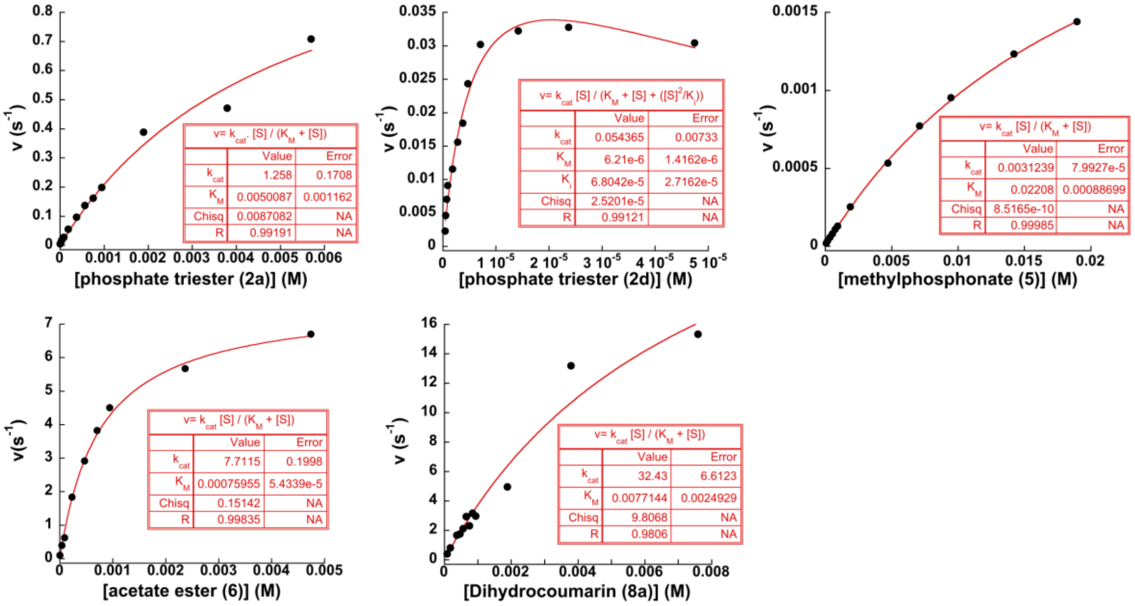

v (s-1)
[phosphate triester (2a)] (M)
v= kcat [S] / (KM + [S])
Value Error
kcat 1.258 0.1708
KM 0.0050087 0.001162
Chisq 0.0087082 NA
R 0.99191 NA
v (s-1)
[phosphate triester (2d)] (M)
v= kcat [S] / (KM + [S] + ([S]2/Ki))
Value Error
kcat 0.054365 0.00733
KM 6.21e-6 1.4162e-6
Ki 6.8042e-5 2.7162e-5
Chisq 2.5201e-5 NA
R 0.99121 NA
v (s-1)
[methylphosphonate (5)] (M)
v= kcat [S] / (KM + [S])
Value Error
kcat 0.0031239 7.9927e-5
KM 0.02208 0.00088699
Chisq 8.5165e-10 NA
R 0.99985 NA
v(s-1)
[acetate ester (6)] (M)
v= kcat [S] / (KM + [S])
Value Error
kcat 7.7115 0.1998
KM 0.00075955 5.4339e-5
Chisq 0.15142 NA
R 0.99835 NA
v (s-1)
[Dihydrocoumarin (8a)] (M)
v= kcat [S] / (KM + [S])
Value Error
kcat 32.43 6.6123
KM 0.0077144 0.0024929
Chisq 9.8068 NA
R 0.9806 NA

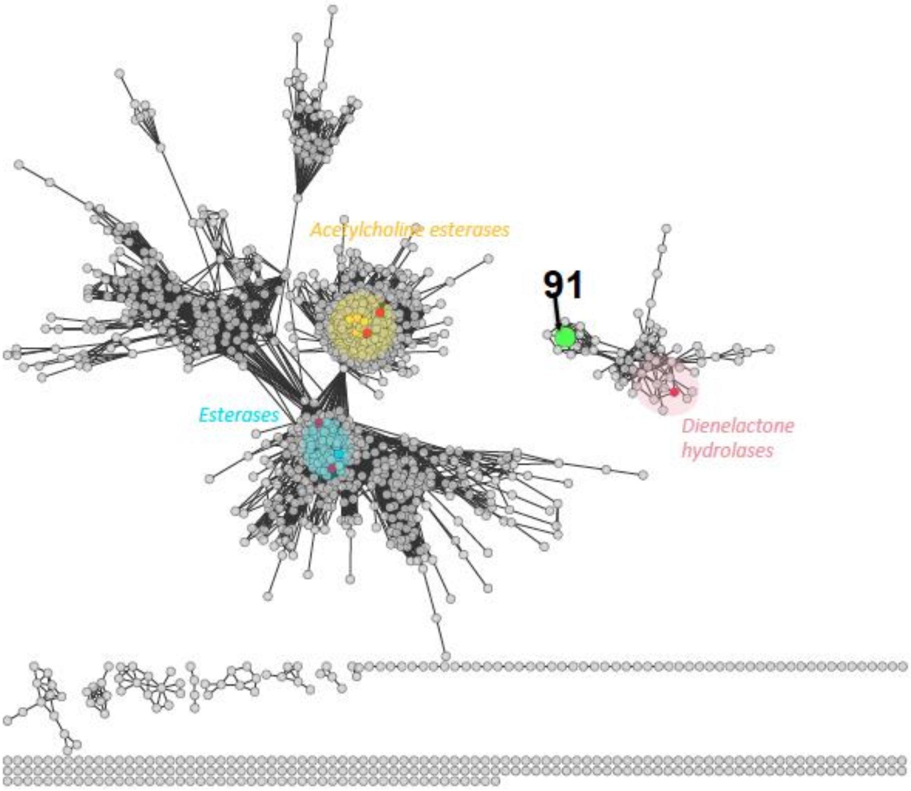

Supplementary Figure 15: Sequence similarity network of α/β hydrolase superfamily members.

1345 sequences from the α/β hydrolase superfamily are represented in this network. Only edges corresponding to similarity scores below E-values of 1 x e^{-19} are shown; the worst edges shown represent a median 30 % identity over an alignment length of 232 residues.

Only three families from the α/β hydrolase superfamily (with experimentally characterised proteins (red nodes)) are represented here: acetylcholine esterases (PF000135) (sequences annotated as such in the Uniprot database are highlighted in yellow), esterases (PF07859) (sequences annotated as such in the Uniprot database are highlighted in blue) and dienelactone hydrolases (PF01738) (highlighted in pink – the red node corresponds to the Uniprot sequence P0A114). Some acetylcholine esterases (yellow nodes) were shown to confer insecticide resistance in insects[4, 5] and an esterase (OpdB) from *Lactobacillus brevis* WCP902 was shown to degrade organophosphate pesticide[6] (blue node).

a

UNIPROT Id	Q4J6Z8	D0VX6	**P83**	Q8GC45	Q93LD7	Q47934	P42084	Q8U8Z6	**P88.1**	**P88.2**	C8C3Z2	Q9S1C6
Q4J6Z8	100											
D0VX6	34.7	100										
**P83**	32.5	43.2	100.0									
Q8GC45	32.5	28.2	34.6	100								
Q93LD7	34.1	29.1	34.7	89.6	100.0							
Q47934	24.6	21.8	24.8	69.8	62.9	100.0						
P42084	7.9	10.3	9.0	9.3	9.4	7.7	100.0					
Q8U8Z6	6.9	11.8	8.1	9.2	8.4	10.3	40.3	100.0				
**P88.1**	8.6	11.7	10.6	10.4	10.2	9.2	13.3	14.6	100.0			
**P88.2**	8.9	9.5	8.1	9.6	8.4	8.3	13.1	16.0	66.9	100.0		
C8C3Z2	8.6	8.3	6.7	8.2	8.4	7.7	16.0	13.5	18.6	21.5	100.0	
Q9S1C6	8.3	8.3	6.7	8.5	8.7	7.7	16.3	13.8	18.8	21.7	98.6	100.0

b

UNIPROT Id	Q93SP1	Q841S6	Q3S3C9	B2Z3X1	Q6DTN5	Q5W503	D8JE85	**P85**	**P86**	G3M1G2	**P87**	**P90**	**P84**
Q93SP1	100.0												
Q841S6	99.7	100.0											
Q3S3C9	98.5	98.8	100.0										
B2Z3X1	98.2	98.5	98.5	100.0									
Q6DTN5	91.7	92.0	91.7	91.6	100.0								
Q5W503	45.0	45.3	45.3	45.6	45.9	100.0							
D8JE85	37.9	37.9	37.9	38.5	38.2	39.6	100.0						
**P85**	21.1	21.1	21.5	21.6	21.1	17.1	16.7	100.0					
**P86**	20.1	20.1	20.5	20.6	20.1	15.6	16.4	68.1	100.0				
G3M1G2	30.2	30.2	30.8	30.2	32.0	32.0	30.0	14.2	14.1	100.0			
**P87**	19.9	19.9	19.5	20.0	20.3	20.0	18.9	14.2	14.8	15.5	100.0		
**P90**	20.2	20.2	20.2	19.7	20.7	21.5	18.2	11.3	11.1	19.7	18.9	100.0	
**P84**	17.9	17.9	17.9	17.4	18.3	19.0	15.8	12.3	12.8	16.8	12.2	28.0	100.0

Supplementary Figure 16: Comparison of sequence identity between reported phosphotriesterases (annotated as such in Uniprot) and our metagenomic hits in the amidohydrolase superfamily (**a**) and MBL superfamily (**b**).
The scale colour reflects the lowest to highest identity percentage (from low values in red to high values in blue). The alignments were performed with ClustalW2 using default settings and displayed using the percentage identity matrix.

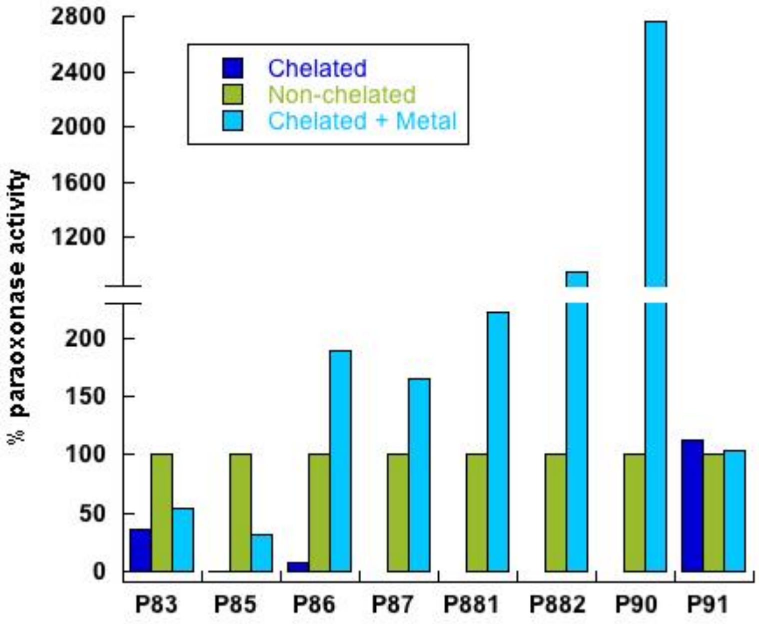

Supplementary Figure 17: Effect of metal removal in phosphotriesterase activity. Initial rates were measured for hydrolysis of triester **2a**. The remaining paraoxonase activities after chelating treatment (blue); without chelating treatment (red) and with chelating treatment then re-incubated with 200 μM $MnCl_2$ are represented. ***Conditions***: [MOPS] = 100 mM, [NaCl] = 150 mM, pH 8.0 at 25°C; [**2a**] = 800 μM.

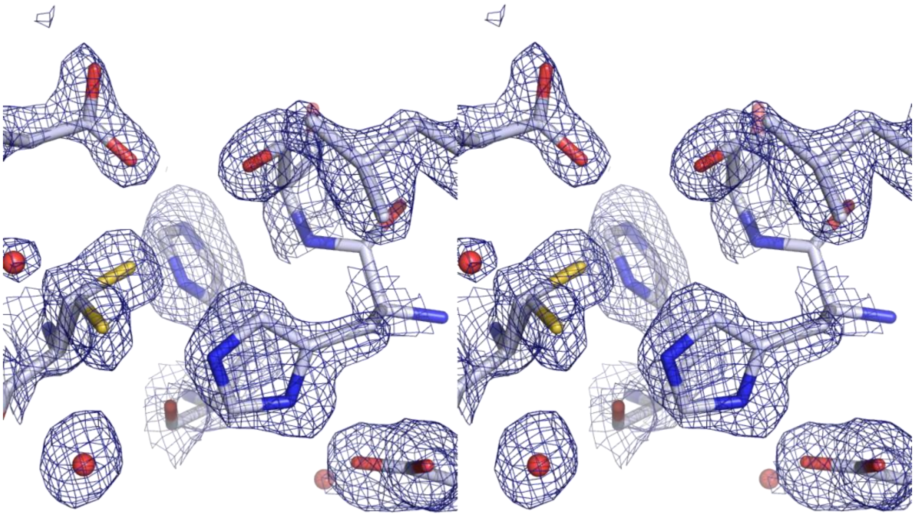

Supplementary Figure 18: Cross-eyed stereo view of the active site of P91. Electron density has been traced as a $2F_{obs}$-F_{calc} -map at 1.5 σ (blue grid), selected amino acids are shown as sticks (grey).

Supplementary Tables

Supplementary Table 1: Metagenomic libraries and their environmental sources.

	Library Name	Size (Number of Variants)	DNA source
1	ENR-M*	23,000	Marine Sludge [7]
2	ENR-G*	25,000	Goose Pond [7]
3	ENR-S*	35,000	Sandy Soil [7]
4	ENR-L*	30,000	Loamy Soil [7]
5	DIR-L	80,000	Loamy Soil [7]
6	SEM	80,000	Vanilla Pods
7	TSA	45,000	Vanilla Pods
8	DIR-MC	500,000	Medium Compost
9	DIR-MC	300,000	Thermophilic Ripe Compost
10	CR2	135,000	Cow Rumen
	Total	1,250,000	

Library sizes were evaluated after transformation of the ligation products between either the sheared metagenomic DNA (libraries 1 to 9) or the digested metagenomic DNA (library 10) with the plasmid pZero-2. The starred libraries (*) were pooled to give the combined library ENR-MSGL that was used for preliminary experiments presented in Supplementary Fig. 3 and 4. These libraries were obtained after enrichment of microorganisms able to grow with specific nitrogen sources[7].

Supplementary Table 2: Metagenomic sequences containing sulfatase or phosphotriesterase hits.

Hit	Closest genome[a] (match % ; % identity)	DNA source[b] - % GC – Insert size (kbp)
**PC32**	*Pseudomonas putida (79 ; 94)*	Soil – 64 – 6.7
**PC35**	*Variovorax paradoxus* (62 ; 90)	Soil - 63 – 3.1
**PC40**	*Paenibacillus sp. (92 ; 81)*	Soil - 52 – 3.1
**BK1**	*Pseudomonas mosselii (91 ; 83)*	Soil - 66 – 2.7
**PC76**	*Variovorax paradoxus (6 ; 80)*	Soils/Vanilla pods - 65 – 2.1
**PC82**	P*eudomonas putida (99; 91)*	Soils/Vanilla pods - 63 – 3.7
**PC83**	no similar sequences	Soils/Vanilla pods - 64 – 2.5
**PC84**	*Uncultured bacterium LAB20* (13 ; 85)	Cow rumen- 65 – 2.7
**PC85**	no similar sequences	Cow rumen - 53 – 2.7
**PC86**	*Herbaspirillum seropedicae (6 ; 76)*	Cow rumen - 60 – 3.1
**PC87**	no similar sequences	Soils/Vanilla pods - 41 – 1.2
**PC88**	*Bradyrhizobium sp.* (25 ; 72)	Soils/Vanilla pods - 59 – 5.2
**PC90**	*Enterobacter asburiae* LF7a (96 ; 85)	Soils/Vanilla pods - 54 – 2.6
**PC91**	*Halorhodospira halophila (4 ; 80)*	Soils/Vanilla pods - 63 - 3.0

[a] Results obtained by submitting the metagenomic sequence to the NCBI nucleotide database (nucleotide collection nr/nt) in 2014;
[b] The DNA source could be unambiguously ascribed to either cow rumen and soils/vanilla pods since the environmental DNA was cloned into pZero-2 using two different restriction sites.
Note that PC32, PC35, PC40 and BK1 were recovered when a subset of the library originating from soil was screened (Supplementary Fig. 4).

Supplementary Table 3: Kinetic parameters for all hydrolases characterised in this study.

Michaelis-Menten parameters (k_{cat}, K_M, k_{cat}/K_M) of the ten hits identified by metagenomic screening (*i.e*. nine phosphotriesterases and one sulfatase (P35)) were obtained for a range of substrates (Supplementary Fig. 12). Purified enzymes (10 nM < [E] < 1μM) were incubated with different substrate concentrations for up to three hours. The observed initial rates v_o were plotted against substrate concentration. The Michaelis-Menten equation (or its modified version accounting for substrate inhibition) or linear regressions (if solubility limits precluded measurement of an entire saturation profile) were used to fit the data using Kaleidagraph. All the plots are shown in Supplementary Fig. 14.

Substrate		P35				P83				P84			
		k_{cat} (s^{-1})	K_M (M)	K_i (M)	k_{cat}/K_M ($s^{-1}.M^{-1}$)	k_{cat} (s^{-1})	K_M (M)	K_i (M)	k_{cat}/K_M ($s^{-1}.M^{-1}$)	k_{cat} (s^{-1})	K_M (M)	K_i (M)	k_{cat}/K_M ($s^{-1}.M^{-1}$)
4-nitrophenyl sulphate	**1a**	9.4 ± 0.3	5.3±0.6 x10^{-5}	3.3±0.3 x10^{-3}	1.7±0.5 x10^{5}	-	-	-	-	-	-	-	-
4-nitrophenyl diethylphosphate	**2a**	-	-	-	-	-	-	-	2.5±0.1 x10^{3}	-	-	-	7.6±0.1
4-nitrophenyl dimethylthiophosphate	**2b**	nd	nd	nd	nd	-	-	-	1.1±0.1 x10^{-1}	-	-	-	-
fluorescein-di(diethylphosphate)	**2d**	nd	nd	nd	nd	3.6±0.1	3.8±0.4 x10^{-6}	-	9.2±1.0 x10^{5}	3.4±0.1 x10^{-3}	6.5±0.4 x10^{-5}	4.4±0.4 x10^{-4}	52±4
4-nitrophenyl phosphate	**3**	2.2±0.1 x10^{-3}	1.4 ± 0.1 x10^{-2}	-	1.6±0.2 10^{-1}	-	-	-	-	-	-	-	-
4-nitrophenyl ethylphosphate	**4**	9.4±0.9 x10^{-4}	0.1±0.2	-	9.4±1.7x 10^{-3}	-	-	-	-	4.2±0.4 x10^{-4}	9.7±2.0 x10^{-3}	-	4.4±1.0 x10^{-2}
4-nitrophenyl methylphosphonate	**5**	-	-	-	2.8±0.1 x10^{1}	-	-	-	-	2.8±0.2 x10^{-3}	9.1±1.1 x10^{-3}	-	3.1±0.4 x10^{-1}
4-nitrophenyl acetate	**6**	-	-	-	-	0.13±0.02	9.6±3.0 x10^{-4}	-	1.4±0.5 x10^{2}	1.1±0.02	2.3±0.1 x10^{-3}	-	4.6±0.2 x10^{2}
s-lactoylglutathion	**7**	-	-	-	-	-	-	-	-	-	-	-	2.2±0.1 x10^{3}
dihydrocoumarin	**8a**	nd	nd	nd	nd	5.3±0.4	2.5±0.4 x10^{-3}	-	2.2±0.4 x10^{3}	-	-	-	2.1±0.1x 10^{3}
γ-butyrolactone	**8b**	nd	nd	nd	nd								
undecanoic-δ-lactone	**8c**	nd	nd	nd	nd	17.0±0.6	3.9±0.4 x10^{-4}	-	4.5±0.5 x10^{4}	-	-	-	-
N-Butyryl-L-homoserine lactone	**$8d_1$**	nd	nd	nd	nd	-	-	-	-	-	-	-	-
N-Hexanoyl-DL-Homoserine lactone	**$8d_2$**	nd	nd	nd	nd	-	-	-	-	-	-	-	-
4-nitroacetaniline	**9**	-	-	-	-	-	-	-	-	-	-	-	-
CENTA	**10**	nd	nd	nd	nd	nd	nd	nd	nd	-	-	-	-

Substrate		P85				P86			P87			
		k_{cat} (s^{-1})	K_M (M)	K_i (M)	k_{cat}/K_M ($s^{-1}.M^{-1}$)	k_{cat} (s^{-1})	K_M (M)	k_{cat}/K_M ($s^{-1}.M^{-1}$)	k_{cat} (s^{-1}))	K_M (M)	K_i (M)	k_{cat}/K_M ($s^{-1}.M^{-1}$)
4-nitrophenyl sulphate	**1a**	-	-	-	-	-	-	-	-	-	-	-
4-nitrophenyl diethylphosphate	**2a**	-	-	-	9.3±0.3 x10^{1}	-	-	1.9±0.2 x10^{2}	5.0±0.3 x10^{-1}	3.7±0.4 x10^{-3}	-	1.3±0.1 x10^{2}
4-nitrophenyl dimethylthiophosphate	**2b**	-	-	-	-	-	-	-	-	-	-	4.3±0.2 x10^{2}
fluorescein-di(diethylphosphate)	**2d**	8.9±0.6 x10^{-3}	9.4±1.6 x10^{-6}	-	9.5±1.7 x10^{2}	4.2±0.2 x10^{-2}	4.4±0.4 x10^{-5}	1.1±0.1 x10^{3}	2.3±0.1	5.0±0.4 x10^{-5}	1.2±0.1 x10^{-4}	4.6±0.5 x10^{4}
4-nitrophenyl phosphate	**3**	-	-	-	-	-	-	-	-	-	-	-
4-nitrophenyl ethylphosphate	**4**	1.0±0.1 x10^{-4}	2.0±0.4 x10^{-2}	-	5.0±1.0 x10^{-3}	1.8±0.1 x10^{-4}	2.1±0.1 x10^{-2}	8.6±0.5 x10^{-3}	3.6±0.2 x10^{-4}	5.3±0.8 x10^{-3}	-	6.8±1.0 x10^{-2}
4-nitrophenyl methylphosphonate	**5**	-	-	-	1.6±0.1 10^{-1}	-	-	1.0±0.1 x10^{-1}	1.0±0.1 x10^{-3}	9.2±1.2 x10^{-3}	-	1.1±0. x10^{-1}
4-nitrophenyl acetate	**6**	-	-	-	4.4±0.1 x10^{1}	-	-	3.1±0.2 x10^{1}	-	-	-	3.9±0.1 x10^{1}
s-lactoylglutathion	**7**	-	-	-	-	-	-	-	-	-	-	-
dihydrocoumarin	**8a**	-	-	-	-	-	-	-	-	-	-	-
γ-butyrolactone	**8b**	-	-	-	-	-	-	-	-	-	-	-
undecanoic-δ-lactone	**8c**	-	-	-	-	-	-	-	-	-	-	-
N-Butyryl-L-homoserine lactone	**$8d_1$**	-	-	-	-	-	-	-	-	-	-	-
N-Hexanoyl-DL-Homoserine lactone	**$8d_2$**	-	-	-	-	-	-	-	-	-	-	-
4-nitroacetaniline	**9**	-	-	-	-	-	-	-	-	-	-	-
CENTA	**10**	-	-	-	-	-	-	-	-	-	-	-

Substrate		P88.1				P88.2				P90			
		k_{cat} (s^{-1})	K_M (M)	K_i (M)	k_{cat}/K_M ($s^{-1}.M^{-1}$)	k_{cat} (s^{-1})	K_M (M)	K_i (M)	k_{cat}/K_M ($s^{-1}.M^{-1}$)	k_{cat} (s^{-1})	K_M (M)	K_i (M)	k_{cat}/K_M ($s^{-1}.M^{-1}$)
4-nitrophenyl sulphate	**1a**	-	-	-	-	-	-	-	-	-	-	-	-
4-nitrophenyl diethylphosphate	**2a**	2.3±0.2	5.3±1.0 x10^{-3}	-	4.2±0.9 x10^{2}	5.9±0.1 x10^{-1}	3.2±0.1 x10^{-3}	-	1.8±0.1 x10^{2}	1.0±0.1 x10^{-1}	2.2±0.2 x10^{-3}	-	4.6±0.6 x10^{1}
4-nitrophenyl dimethylthiophosphate	**2b**	3.7±0.3 x10^{-2}	1.3±0.3 x10^{-3}	-	2.8±0.7 x10^{1}	3.7±0.5 x10^{-3}	1.9±0.5 x10^{-3}	-	2.0±0.6	4.0±0.5 x10^{-4}	1.1±0.3 x10^{-3}	-	3.8±1.2x 10^{-1}
fluorescein-di(diethylphosphate)	**2d**	7.8±0.4* x10^{-2}	3.1±0.4 x10^{-5}	4.0±0.6 x10^{-4}	2.5±0.3 x10^{3}	3.5±0.3 x10^{-2}	3.1±0.5 x10^{-5}	1.5±0.3 x10^{-4}	1.1±0.2 x10^{3}	3.7±1.4 x10^{-2}	2.3±1.0 x10^{-4}	5.3±2.4 x10^{-5}	1.6±0.9 x10^{2}
4-nitrophenyl phosphate	**3**	-	-	-	-	-	-	-	-	-	-	-	-
4-nitrophenyl ethylphosphate	**4**	-	-	-	-	-	-	-	-	-	-	-	-
4-nitrophenyl methylphosphonate	**5**	-	-	-	-	1.8±0.2 x10^{-3}	1.8±0.4 x10^{-2}	-	1.0±0.3 x10^{-1}	2.7±0.1 x10^{-3}	3.5±0.2 x10^{-3}	-	7.7±0.5 x10^{-1}
4-nitrophenyl acetate	**6**	-	-	-	5.5±0.2 x10^{2}	3.9±0.1 x10^{-2}	2.9±1.2 x10^{-3}	-	1.3±0.7 x10^{1}			-	1.1±0.2 x10^{2}
s-lactoylglutathion	**7**	-	-	-	-	-	-	-	-	-	-	-	2.2±0.1 x10^{3}
dihydrocoumarin	**8a**	-	-	-	-	-	-	-	-	-	-	-	6.3±0.2 x10^{3}
γ-butyrolactone	**8b**	-	-	-	-	-	-	-	-	-	-	-	-
undecanoic-δ-lactone	**8c**	-	-	-	-	-	-	-	-	-	-	-	-
N-Butyryl-L-homoserine lactone	**8d$_1$**	-	-	-	-	-	-	-	-	-	-	-	-
N-Hexanoyl-DL-Homoserine lactone	**8d$_2$**	-	-	-	-	-	-	-	-	-	-	-	-
4-nitroacetaniline	**9**	-	-	-	-	-	-	-	-	-	-	-	-
CENTA	**10**	nd	nd	nd	nd	nd	nd	nd	nd	-	-	-	-

Substrate		P91		
		k_{cat} (s^{-1})	K_M (M)	k_{cat}/K_M ($s^{-1}.M^{-1}$)
4-nitrophenyl sulphate	**1a**	-	-	-
4-nitrophenyl diethylphosphate	**2a**	1.3±0.2	5.0±1.2 x10^{-3}	2.5±0.7 x10^{2}
4-nitrophenyl dimethylthiophosphate	**2b**	-	-	-
fluorescein-di(diethylphosphate)	**2d**	5.4±0.7 x10^{-2}	6.2±1.4 x10^{-6}	8.7±2.3 x10^{3}
4-nitrophenyl phosphate	**3**	-	-	-
4-nitrophenyl ethylphosphate	**4**	-	-	-
4-nitrophenyl methylphosphonate	**5**	3.1±0.1 x10^{-3}	2.2±0.1 x10^{-2}	1.4±0.7 x10^{-1}
4-nitrophenyl acetate	**6**	7.7±0.2	7.6±0.5 x10^{-4}	1.4±0.1 x10^{4}
s-lactoylglutathion	**7**	-	-	-
dihydrocoumarin	**8a**	3.2±0.7 x10^{1}	7.7±2.5 x10^{-3}	4.2±1.6 x10^{3}
γ-butyrolactone	**8b**			
Undecanoic-δ-lactone	**8c**	-	-	-
N-Butyryl-L-homoserine lactone	**8d$_1$**	-	-	-
N-Hexanoyl-DL-Homoserine lactone	**8d$_2$**	-	-	-
4-nitroacetaniline	**9**	-	-	-
CENTA	**10**	nd	nd	nd

'nd': not determined. '-': no product was detected with 1 μM of enzyme and 1 mM of substrate over 3 hours (over a background control in the absence of enzyme). This suggests an upper limit of such activities of approximately 10-fold below the lowest activity observed here (5x10^{-3} M^{-1}s^{-1} for P85) for substrates with *p*-nitrophenol leaving groups.

Conditions: [MOPS] = 100 mM, [NaCl] =150 mM, pH 8.0 at 25 °C. For lactones 8c: [Bicine] = 2.5 mM, [NaCl] = 200 mM, [cresol purple] = 0.3 mM, pH 8.3 at 25 °C. We estimate the detection limit of the lactonase activity assay ~10 s^{-1}M^{-1} (k_{cat}/K_M) as previously reported[8].

Supplementary Table 4: Rate enhancements and catalytic proficiencies of the metagenomic hits

	Substrate	k_{cat}/k_{uncat} [a]	$k_{cat}/K_M/k_{uncat}$ (M^{-1})[b]	$k_{cat}/K_M/k_w$ [c]
P35*	**1a**	$8.6x10^{9}$	$1.5x10^{14}$	$8.5x10^{15}$
P83	**2a**	-	$5.7x10^{10}$	$3.1x10^{12}$
P84	**2a**	-	$1.7x10^{8}$	$9.5x10^{9}$
P85	**2a**	-	$2.1x10^{9}$	$1.2x10^{11}$
P86	**2a**	-	$4.3x10^{9}$	$2.4x10^{11}$
P87	**2a**	$1.1x10^{7}$	$3.0x10^{9}$	$1.6x10^{11}$
P881	**2a**	$5.3x10^{7}$	$9.5x10^{9}$	$5.3x10^{11}$
P882	**2a**	$1.3x10^{7}$	$4.1x10^{9}$	$2.3x10^{11}$
P90	**2a**	$2.3x10^{6}$	$1.0x10^{9}$	$5.8x10^{10}$
P91	**2a**	$3.0x10^{7}$	$5.7x10^{9}$	$3.1x10^{11}$

The rate of the uncatalysed hydrolysis (k_{uncat}) of phosphate triester **2a** was measured at 25 °C in MOPS (100 mM, containing NaCl 150 mM, pH 8): k_{uncat} (**2a**) = 4.4 x 10^{-8} s^{-1}.

*Assuming the rate of uncatalysed hydrolysis of sulfate monoester **1a** being pH-independent between pH 4 and 12; k_{uncat} (**1a**) = 1.1 x 10^{-9} s^{-1}.[9]

[a] First-order rate enhancement; "-": k_{cat} values not accessible from linear fits.

[b] Catalytic proficiencies

[c] Second-order rate enhancement; $k_{uncat} = k_w$ x $[H_2O]$, in which $[H_2O]$ = 55 M.

Supplementary Table 5: Mann-Whitney test comparing catalytic parameters of metagenomic hits and organophosphate-degrading enzymes.

	k_{cat}/K_M			k_{cat}			K_M		
	OPH	Hits	Pro	OPH	Hits	Pro	OPH	Hits	Pro
OPH	-	$8x10^{-5}$	0.01	-	$5x10^{-2}$	0.1	-	$2x10^{-3}$	$2x10^{-3}$
Hits	-	-	0.1	-	-	0.8	-	-	0.1
Pro	-	-	-	-	-	-	-	-	-

Using a Mann-Whitney U test, P-values indicated that our hits' catalytic parameters are more likely to be dissimilar to the catalytic parameters of OPHs than to promiscuous (prom) enzymes. 'OPH' (organophosphate hydrolases) summarises phosphotriesterase recovered from bacteria isolated from a pesticide-polluted environment and therefore assigned as enzymes evolved specifically for triester hydrolysis. 'Pro' summarises enzymes for which phosphotriesterase activity was shown to be a side activity.

Supplementary Table 6: Multiple turnover organophosphate pesticides degrading enzymes from the literature.

Subgroup	Uniprot ID	Organism	Superfamily[a]
**Opd**	Q93LD7	*Rhizobium radiobacter*	Amidohydrolase
**Opd**	B3GN95	*Sphingomonas sp. JK1*	Amidohydrolase
**Opd**	Q5UB52	*Flavobacterium sp. MTCC 2495*	Amidohydrolase
**Opd**	Q8VLR0	*Flavobacterium balustinum*	Amidohydrolase
**Opd**	P0A434	*Pseudomonas diminuta*	Amidohydrolase
**Opd**	M29593	*Flavobacterium sp. ATCC 27551*	Amidohydrolase
**Opd**	Q93LD7	*Agrobacterium radiobacter (P230)*	Amidohydrolase
**PLL (DrPLL)**	Q9RVU2	*Deinococcus Radiodurans*	Amidohydrolase
**PLL (VmutPLL)**	F0QXN6	*Vulcanisaeta moutnovskia*	Amidohydrolase
**PLL (SacPox)**	Q4J6Z8	*Sulfolobus acidocaldarius*	Amidohydrolase
**PLL (GsP)**	D0VX06	*Geobacillus stearothermophilus*	Amidohydrolase
**PLL (SsoPox)**	Q97VT7	*Sulfolobus solfataricus*	Amidohydrolase
**PLL (SisLac)**	C4KKZ9	*Sulfolobus islandicus*	Amidohydrolase
**P83**		Metagenome (this study)	Amidohydrolase
**P88_1**		Metagenome (this study)	Amidohydrolase
**P88_2**		Metagenome (this study)	Amidohydrolase
**IMH**	C8C3Z2	*Arthrobacter sp.*	Amidohydrolase
**MPH**	Q6DTN5	*Burkholderia sp. FDS-1*	MBL
**MPH**	Q1WDQ6	*Sphingomonas sp. Dsp-2*	MBL
**MPH**	Q52I83	*Burkholderia cepacia*	MBL
**MPH**	Q693X3	*Achromobacter sp. mp-2*	MBL
**MPH**	B2Z3X1	*Ochrobactrum sp. M231*	MBL
**MPH**	Q693W8	*Brucella sp. mp-7*	MBL
**MPH**	Q0PJ60	*Stenotrophomonas sp. OP-1*	MBL
**MPH**	Q841S6	*Pseudomonas sp. (strain WBC-3)*	MBL
**MPH**	Q93SP1	*Pseudomonas putida*	MBL
**MPH**	Q693X2	*Ochrobactrum sp. mp-3*	MBL
**MPH**	B2BHT5	*Pseudomonas stutzeri*	MBL
**MPH**	Q693X4	*Pseudaminobacter sp. mp-1*	MBL
**Ophc2**	B2ZF61	*Stenotrophomonas sp. SMSP-1*	MBL
**Ophc2**	Q5W503	*Pseudomonas pseudoalcaligenes*	MBL
**P84**		Metagenome (this study)	MBL
**P85**		Metagenome (this study)	MBL
**P86**		Metagenome (this study)	MBL
**P87**		Metagenome (this study)	MBL
**P90**		Metagenome (this study)	MBL
**OphB**	Q25C66	*Burkholderia sp. NF100*	-
**OphB**	A4ZYB5	*Burkholderia sp. JBA3*	-
**OpdB**	C0K3M4	*Lactobacillus brevis WCP902*	α/β hydrolase
**LcaE7**	Q25252	*Lucilia cuprina*	α/β hydrolase
**FE4**	P35502	*Myzus persicae*	α/β hydrolase
**Ld AChE**	Q27677	*Leptinotarsa decemlineata*	α/β hydrolase
**Dm AChE**	CG17907	*Drosophila melanogaster*	α/β hydrolase
**Ag AChE-1**	AAM94376	*Aphis gossypii*	α/β hydrolase
**P91**		*Metagenome (this study)*	α/β hydrolase
**GpdQ**	A8HR62	*Enterobacter aerogenes*	Calcineurin-like phosphoesterase
**OpaA**	P77814	*Alteromonas haloplanktis*	Xaa-Pro dipeptidase
**OpaA**	Q44238	*Alteromonas sp. JD6.5*	Xaa-Pro dipeptidase
**AMPP**	P15034	*Escherichia coli*	Xaa-Pro dipeptidase
**OpdE**	H6BF92	*Enterobacter sp.*	DNase I-like
**BcPMH**	Q45087	*Burkholderia caryophylli*	Alkaline phosphatase
**PON1**	P27169	*Homo sapiens*	β-propeller
**DFP**	Q7SIG4	*Loligo vulgaris*	β-propeller

[a]Superfamily defined as PFAM clan.

The metagenomic hits found in this study are highlighted in green. Lines highlighted in blue (OPH) and purple (Promiscuous) are proteins used for comparison with our triesterase hits (Fig. 4b). Details of catalytic efficiencies for highlighted enzymes are listed in Supplementary Table 7.

Supplementary Table 7: Phosphotriesterase activity of bacterial organophosphate-pesticide degrading enzymes described in the literature.

	Name	Microorganism	k_{cat}/K_M ($s^{-1}M^{-1}$)	K_M (mM)	Sub*	Ref
	P83	SCV library	3x10^{3}	>5.0	**2a**	This study
	P84	SCV library	8	>5.0	**2a**	This study
	P85	SCV library	9x10^{1}	>5.0	**2a**	This study
	P86	SCV library	2x10^{2}	>5.0	**2a**	This study
Hits	P87	SCV library	1x10^{2}	3.7	**2a**	This study
	P88.1	SCV library	4x10^{2}	5.3	**2a**	This study
	P88.2	SCV library	2x10^{2}	3.2	**2a**	This study
	P90	SCV library	5x10^{1}	2.2	**2a**	This study
	P91	SCV library	3x10^{2}	5.0	**2a**	This study
	PdPTE	*Pseudomonas. diminuta*	9x10^{7}	4.8x10^{-2}	**2a**	[8]
	Opd	*Flavobacterium sp. ATCC 27551*	4x10^{7}	1x10^{-2}	**2a**	[5]
	OpdA	*Agrobacterium radiobacter (P230)*	2x10^{7}	4.1x10^{-2}	**2a**	[10]
	IMH	*Arthrobacter sp. scl-2*	1x10^{5}	2.8x10^{-2}	**2b**	[11]
OPH	MPH	*Burkholderia cepacia*	2x10^{4}	8.6x10^{-2}	**2a**	[12]
	MPH	*Ochrobactrum sp. M231*	6x10^{4}	7.6x10^{-2}	**2b**	[12]
	MPH	*Pseudomonas sp. (strain WBC-3)*	1x10^{6}	3.7x10^{-2}	**2b**	[12]
	Ophc2	*Pseudomonas pseudoalcaligenes*	3x10^{3}	2.1x10^{-2}	**2b**	[12]
	DrPLL	*Deinococcus Radiodurans*	3x10^{1}		**2a**	[13]
	VmutPLL	*Vulcanisaeta moutnovskia*	4x10^{2}	2.8	**2b**	[14]
	SacPox	*Sulfolobus acidocaldarius*	1x10^{3}	2.8x10^{-1}	**2b**	[15]
Pro	GsP	*Geobacillus stearothermophilus*	5x10^{1}	2.1	**2a**	[16]
	SsoPox	*Sulfolobus solfataricus*	5x10^{2}	2.4	**2a**	[17]
	SisLac	*Sulfolobus islandicus*	4x10^{3}	1.7	**2b**	[18]
	GpdQ	*Enterobacter aerogenes*	8x10^{2}	6.0x10^{-2}	**2a**	[19]
	OpaA	*Alteromonas sp. JD6.5*	1x10^{2}	9.5	**2a**	[12]
	BcPMH	*Burkholderia caryophilli*	2x10^{-2}	>2.4	**2a**	[9]

*Substrates: **2a**=paraoxon ; **2b**=methylparathion; see Supplementary Fig. 12.

Reported rates were measured at 25-30 °C, pH 7.0 – 8.0. Values reported here were used to compare the metagenomic hits to known enzymes isolated from polluted environments (OPH) and to enzymes endowed with triesterase promiscuous activity (Pro). *Bc*PMH was not considered for the comparison of catalytic efficiencies in Table 1.

Supplementary Table 8: Data collection and refinement statistics

	P91 (PDB: 4ZI5)
**Data collection**	
Space group	P 2_1 2_1 2_1
Cell dimensions	
a, *b*, *c* (Å)	55.61, 73.41, 113.06
α, β, γ (°)	90.00, 90.00, 90.00
Resolution (Å)	61.57 (1.70)
R_{merge}	0.06 (0.45)
R_{pim}	0.033 (0.298)
I / σI	12.5 (2.02)
Completeness (%)	87.6 (55.6)
$CC_{1/2}$	1.00 (0.85)
Redundancy	3.8 (2.8)
**Refinement**	
Resolution (Å)	61.57-1.702 (1.746-1.702)
No. reflections	42939
R_{work} / R_{free}	0.183/0.207 (0.195/0.218)
No. atoms	4157
Protein	3688
Ions (Mg^{2+}/Cl^-)	2/1
Water	466
B-factors (mean)	20.2
Protein	19.9
Ions (Mg^{2+}/Cl^-)	25.1/62.6
Water	27.1
R.m.s. deviations	
Bond lengths (Å)	0.010
Bond angles (°)	1.389

*Values in parentheses are for the highest-resolution shell.

Supplementary Note 1

Analysis of the gene context of the selected hits.

The context of the selected hits in the plasmid with inserts contributed to a tentative assignment of possible 'native' (or physiological) activities to these new genes. Metabolic pathways are often organised as operons or clusters in bacteria and archea, thus the neighbouring genes may be provide information about their original function[20]. However, the size of the DNA on either side of the identified open reading frame (ORF) in our plasmid-based libraries is small (total insert size: between 1-5 kbp). Compared to metagenomic selections that involve DNA constructs with larger inserts (e.g. fosmid, cosmid or BACs-based libraries), the following considerations are necessarily somewhat speculative.

Each metagenomic sequence was submitted to ORFfinder (http://www.ncbi.nlm.nih.gov/gorf/gorf.html) that enables the identification of potential coding genes (the default parameters consider start and end of an ORF based on the canonical methionine start codon and the three stop codons). For each sequence submitted several ORFs were predicted. However, we considered only potential ORFs in cases where the corresponding proteins were homologs to known proteins in the non-redundant (nr) databases. Each ORF that passed this test was blasted against the nr database from NCBI and the closest homolog was reported (with the highest E-value considered in the resulting sequences $<e^{-6}$ suggesting true homologs). Each selected ORF was also submitted to the PFAM domain database to assign a superfamily and complement the annotations from the nr database, which is presumed to be more prone to missannotations.

Each metagenomic sequence was also tested by nucleotide BLAST (http://blast.ncbi.nlm.nih.gov/Blast.cgi) to identify potential genomes with similar genetic sequences (Supplementary Table 2). If significantly close organisms were identified (i.e. for PC88 and PC90, Supplementary Table 2), their genomic sequence and the ORFs contained therein were directly compared to those from the metagenomic sequence.

Note: PCXX refers to the full metagenomic insert sequence; *p*XX refers to the gene coding for the triesterase.

• **Sequence PC83**

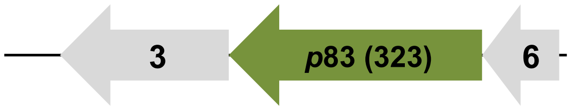

**Database**	**Gene 3**	**Gene 6**
pBlast	ABC transporter	Carnitine dehydratase
PFAM (Clan)	Periplasmic binding protein	-

The sequence context of ***p*83** contains a gene predicted to code for an ABC transporter (gene 3) likely to be implicated in the internalisation of extracellular molecules. Another neighbouring gene (gene 6) is related to Carnitine dehydrogenases and AcylCoA transferases (with sequence identity <50%), although alignment scores suggested that gene 6 is not present in full length in the metagenomic insert. Nonetheless these results could suggest that the genes are involved in the metabolism of lipids.

• **Sequence PC84**

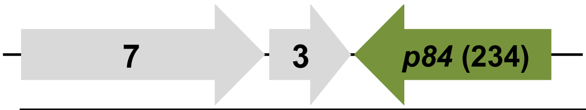

**Database**	**Gene 7**	**Gene 3**
pBlast	Penicillin-binding protein	Membrane protein
PFAM (Clan)	Serine beta lactamase-like	-

The gene ***p84*** was located in a metagenomic sequence comprising two other genes: gene 7 predicted to code for a penicillin-binding protein and gene 8 predicted as a membrane protein. Gene 7 predicted function suggests that the

genes in the sequence PC84 may be involved in the synthesis of peptidoglycan.

• Sequence PC85

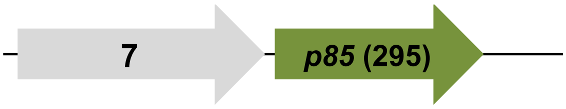

Databa se	Gene 7
pBlast	Penicillin-binding protein
PFAM (Clan)	Serine beta lactamase-like

The gene ***p*85** was co-selected with a gene (gene 7) predicted to be part of the serine β-lactamase superfamily again suggesting a possible role in peptidoglycan synthesis.

• Sequence PC86

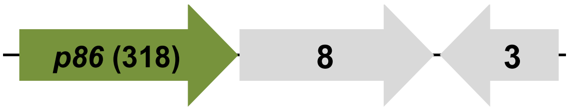

Database	Gene 8	Gene 3
pBlast	Radical SAM protein	ABC transporter
PFAM (Clan)	Radical SAM	ABC transporter

The gene ***p*86** was co-selected with a gene coding for radical SAM protein (gene 8) and a second gene coding for an ABC transporter (gene 3). Gene 8 codes for a 2-domain protein: a SAM domain and a 4Fe-4S domain. Radical SAM proteins were shown to catalyse various reactions such as methylation,

isomerisation, anaerobic oxidation. The lack of additional information precludes further speculation on the physiological function of the protein encoded in the sequence PC86.

• **Sequence PC87**

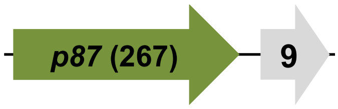

Database	Gene 9
pBlast	K^+/H^+ antiporter
PFAM (Clan)	-

The gene ***p87*** was associated with a small gene (gene 9) related to the sequence of a subunit of a potassium transporter. The lack of additional information precludes further speculation on the physiological function of the proteins encoded in the PC87 metagenomic sequence.

• **Sequence PC88**

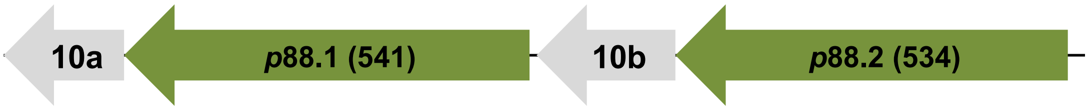

Database	Gene 10a	Gene 10b
pBlast	Cobalamin synthesis protein	Cobalamin synthesis protein
PFAM (Clan)	Cobalamin synthesis CobW	Cobalamin synthesis CobW

The insert **PC88** contained 4 genes: the two genes ***p88.1*** and ***p88.2*** separated by genes (10a and 10b) predicted to code for cobalamin synthesis proteins containing nucleotide-binding domains that could correspond to ATP

binding domains. Interestingly proteins belonging to the amidohydrolase superfamily (as P88.1 and P88.2) have been identified to be involved in the biosynthesis pathway of cobalamin in Archea[21]. The metagenomic insert seems organised as a duplicated system: the motif comprising a amidohydrolase gene (p88) and a cobalamin synthesis gene is repeated. The two amidohydrolase genes are closely related with 66% sequence identity (protein level), whereas the two cobalamin synthesis protein share 58% sequence identity (protein level – on the parts that could be aligned) suggesting a relatively recent duplication event that has shaped the genetic organisation of the metagenomic sequence PC88.

When the nucleotide sequence PC88 is was compared by BLAST against the nt collection database, 25% of the metagenomic sequence align with parts of the genome from *Bradyrhizobium sp*. ORS278. This region also contains genes coding for putative amidohydrolases and cobalamin synthesis proteins. Interestingly the amidohydrolase from *Bradyrhizobium sp*. ORS278 is closely related to P88.1 and P88.2 (~61% sequence identity).

Overall, the analysis of the genes constituting the sequence PC88 and their comparison with similar genomic organisation seem to suggest a role in the synthesis of the cobalamin cofactor.

• Sequence PC90

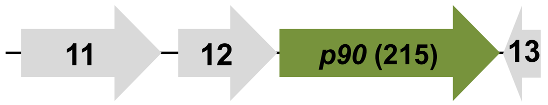

Database	Gene 11	Gene 12	Gene 13
pBlast	Transpeptidase	Hypothetical protein	Aminotransferase
PFAM (Clan)	Transpeptidase YkuD	Peptidase M15	Aminotransferase

The gene ***p*90** was isolated with three other genes coding for proteins related to L,D-transpeptidase (gene 11), peptidase M15 (gene 12) and aminotransferase (gene 13).

The metagenomic insert sequence PC90 is similar (85% sequence identity) to a genomic sequence from *Enterobacter asburiae* LF7a, suggesting that the sequence PC90 originates from a strain closely related to this organism. A similar genetic organisation (i.e. same genes) is found in the genome of *Enterobacter asburiae* LF7a. Interestingly the β-lactamase sequences from both genetic sequences are 93% identical. The transpeptidase from *Enterobacter asburiae* LF7a is annotated as murein transpeptidase. Thus in both PC90 and *Enterobacter asburiae* LF7a the genes 11, 12 and 13 seem to code for proteins with activities with a link to peptidoglycan synthesis.

• Sequence PC91

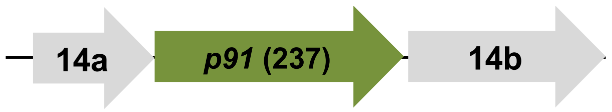

Database	Gene 14a	Gene 14b
pBlast	MFS transporter	4-hydroxybenzoate transporter
PFAM (Clan)	-	Major Facilitator Superfamily

Two permease genes or Major Facilitator Superfamily (MFS) transporters (14a and 14b) surround the gene p91. The gene 14b is related to 4-hydroxybenzoate transporters (36% identity at the protein level). 4-hydroxybenzoate is a precursor of the β-ketoadipate pathway[22], which comprises dienelactones. Thus the sequence PC91 contains genes likely to be involved in the β-ketoadipate pathway, widely distributed in soil bacteria. Aromatic compounds are degraded by this pathway, feeding the Krebs cycle downstream[22].

Supplementary References

1. Gabor, E.M., Alkema, W.B. & Janssen, D.B. Quantifying the accessibility of the metagenome by random expression cloning techniques. *Environ. Microbiol.* **6**, 879-886 (2004).
2. Kintses, B. et al. Picoliter cell lysate assays in microfluidic droplet compartments for directed enzyme evolution. *Chem. Biol.* **19**, 1001-1009 (2012).
3. Jones, T.B. Electromechanics of Particles. *Cambridge University Press* (1995).
4. Jackson, C.J. et al. Structure and function of an insect alpha-carboxylesterase (alphaEsterase7) associated with insecticide resistance. *Proc. Natl. Acad. Sci. U. S. A.* **110**, 10177-10182 (2013).
5. Russell, R.J. et al. The evolution of new enzyme function: lessons from xenobiotic metabolizing bacteria versus insecticide-resistant insects. *Evol. Appl.* **4**, 225-248 (2011).
6. Islam, S.M. et al. Organophosphorus hydrolase (OpdB) of Lactobacillus brevis WCP902 from kimchi is able to degrade organophosphorus pesticides. *J. Agric. Food Chem.* **58**, 5380-5386 (2010).
7. Gabor, E.M., de Vries, E.J. & Janssen, D.B. Construction, characterization, and use of small-insert gene banks of DNA isolated from soil and enrichment cultures for the recovery of novel amidases. *Environ. Microbiol.* **6**, 948-958 (2004).
8. Afriat-Jurnou, L., Jackson, C.J. & Tawfik, D.S. Reconstructing a missing link in the evolution of a recently diverged phosphotriesterase by active-site loop remodeling. *Biochemistry* **51**, 6047-6055 (2012).
9. van Loo, B. et al. An efficient, multiply promiscuous hydrolase in the alkaline phosphatase superfamily. *Proc Natl Acad Sci USA* **107**, 2740-2745 (2010).
10. Ely, F. et al. The organophosphate-degrading enzyme from Agrobacterium radiobacter displays mechanistic flexibility for catalysis. *Biochem. J.* **432**, 565-573 (2010).
11. Li, R. et al. An isofenphos-methyl hydrolase (Imh) capable of hydrolyzing the P–O–Z moiety of organophosphorus pesticides containing an aryl or heterocyclic group. *Appl. Miocrobiol. Biotechnol.* **94**, 1553-1564.
12. Chino-Flores, C. et al. Isolation of the opdE gene that encodes for a new hydrolase of Enterobacter sp. capable of degrading organophosphorus pesticides. *Biodegradation* **23**, 387-397 (2012).
13. Meier, M.M. et al. Molecular engineering of organophosphate hydrolysis activity from a weak promiscuous lactonase template. *J. Am. Chem. Soc.* **135**, 11670-11677 (2013).
14. Kallnik, V. et al. Characterization of a phosphotriesterase-like lactonase from the hyperthermoacidophilic crenarchaeon Vulcanisaeta moutnovskia. *J. Biotechnol.* **190**, 11-17 (2014).
15. Bzdrenga, J. et al. SacPox from the thermoacidophilic crenarchaeon Sulfolobus acidocaldarius is a proficient lactonase. *BMC Res. Notes* **7**, 333 (2014).
16. Hawwa, R., Aikens, J., Turner, R.J., Santarsiero, B.D. & Mesecar, A.D. Structural basis for thermostability revealed through the identification

and characterization of a highly thermostable phosphotriesterase-like lactonase from Geobacillus stearothermophilus. *Arch. Biochem. Biophys.* **488**, 109-120 (2009).
17. Hiblot, J., Gotthard, G., Chabriere, E. & Elias, M. Characterisation of the organophosphate hydrolase catalytic activity of SsoPox. *Sci. Rep.* **2**, 779 (2012).
18. Hiblot, J., Gotthard, G., Chabriere, E. & Elias, M. Structural and enzymatic characterization of the lactonase SisLac from Sulfolobus islandicus. *PLoS One* **7**, e47028 (2012).
19. Ghanem, E.L., Y.; Xu, C.; Raushel, F.M. Characterization of a phosphodiesterase capable of hydrolyzing EA 2192, the most toxic degradation product of the nerve agent VX. *Biochemistry* **46**, 9032-9040 (2007).
20. Zhao, S. et al. Prediction and characterization of enzymatic activities guided by sequence similarity and genome neighborhood networks. *eLife* **e03275** (2014).
21. Woodson, J.D. & Escalante-Semerena, J.C. CbiZ, an amidohydrolase enzyme required for salvaging the coenzyme B12 precursor cobinamide in archaea. *Proc. Natl. Acad. Sci. U. S. A.* **101**, 3591-3596 (2004).
22. Harwood, C.S. & Parales, R.E. The β-ketoadipate pathway and the biology of self-identity. *Annu. Rev. Microbiol.* **50**, 553-590 (1996).
